# The MICALs are a Family of F-actin Dismantling Oxidoreductases Conserved from Drosophila to Humans

**DOI:** 10.1038/s41598-017-17943-5

**Published:** 2018-01-17

**Authors:** Heng Wu, Hunkar Gizem Yesilyurt, Jimok Yoon, Jonathan R. Terman

**Affiliations:** 1 Departments of Neuroscience and Pharmacology, Harold C Simmons Comprehensive Cancer Center, The University of Texas Southwestern Medical Center, Dallas, TX 75390 USA; 2 Present Address: Drug Development Center, SK biopharmaceuticals Co. Ltd., Seongnam, 13494 Korea

## Abstract

Cellular form and function – and thus normal development and physiology – are specified via proteins that control the organization and dynamic properties of the actin cytoskeleton. Using the Drosophila model, we have recently identified an unusual actin regulatory enzyme, Mical, which is directly activated by F-actin to selectively post-translationally oxidize and destabilize filaments – regulating numerous cellular behaviors. Mical proteins are also present in mammals, but their actin regulatory properties, including comparisons among different family members, remain poorly defined. We now find that each human MICAL family member, MICAL-1, MICAL-2, and MICAL-3, directly induces F-actin dismantling and controls F-actin-mediated cellular remodeling. Specifically, each human MICAL selectively associates with F-actin, which directly induces MICALs catalytic activity. We also find that each human MICAL uses an NADPH-dependent Redox activity to post-translationally oxidize actin’s methionine (M) M44/M47 residues, directly dismantling filaments and limiting new polymerization. Genetic experiments also demonstrate that each human MICAL drives F-actin disassembly *in vivo*, reshaping cells and their membranous extensions. Our results go on to reveal that MsrB/SelR reductase enzymes counteract each MICAL’s effect on F-actin *in vitro* and *in vivo*. Collectively, our results therefore define the MICALs as an important phylogenetically-conserved family of catalytically-acting F-actin disassembly factors.

## Introduction

Single actin proteins have the ability to assemble together into long chains or filaments and it is this actin polymerization that underlies cellular structure and behavior^1,2^. A critical goal therefore is to understand the factors that specify the organization and dynamic properties of actin. Over the years a number of proteins have been identified that directly promote actin filament (F-actin) assembly, stability, and generate branched actin networks^1–3^. These actin regulatory proteins include some of the best studied such as bundling proteins like fascin and alpha-actinin, nucleators like formins and Arp2/3, barbed-end/pointed-end capping proteins like CapZ and tropomodulin, and actin assembly proteins like profilin^3–6^.

In contrast to these positive effectors of actin filament assembly, the actin cytoskeleton is also regulated by proteins that negatively influence its stability and organization^7–10^. Recently, while using genetic and biochemical assays in Drosophila we identified a new actin disassembly protein, Mical, that directly binds F-actin and disassembles actin filaments *in vitro* and *in vivo*^11^. Interestingly, our work also identified that Mical is a flavoprotein monooxygenase/hydroxylase enzyme that associates with flavin adenine dinucleotide (FAD) and uses the co-enzyme nicotinamide adenine dinucleotide phosphate (NADPH) in oxidation-reduction (redox) reactions^12,13^. Further investigating this unusual enzyme revealed that Mical uses actin filaments as a direct substrate, selectively binding and stereospecifically oxidizing two conserved amino acids (methionine (Met) 44 and 47) within the pointed-end of actin to dismantle actin filaments and limit F-actin re-assembly^13^. Likewise, investigations revealed that this Mical-mediated post-translational actin regulatory process is reversible by a specific methionine sulfoxide reductase enzyme called SelR/MsrB^14,15^ – and that this reversible redox actin regulatory system controls multiple behaviors in different tissues including cellular remodeling, motility, process elongation, axon guidance, synaptogenesis, and muscle morphology/function^14^. Mical is therefore a new type of F-actin disassembly factor, one that works through the covalent modification of actin – and one that we have also recently linked to working with important non-covalent regulators of actin such as cofilin^16^ and classical receptor-mediated signal transduction pathways^17^.

**Figure 1:**
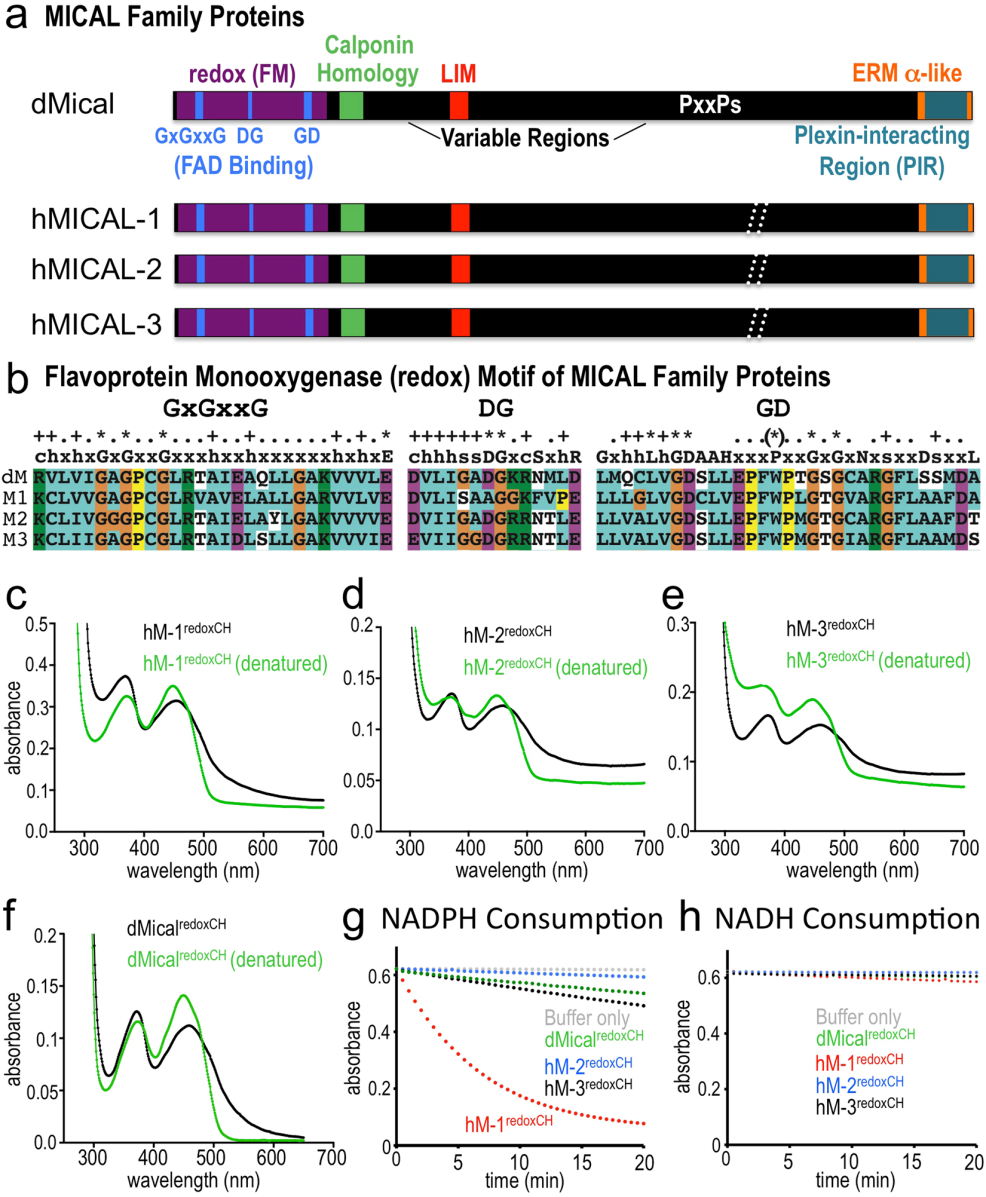
Each human MICAL family member (MICAL-1, MICAL-2, and MICAL-3) is a flavoenzyme that consumes NADPH. (**a**) Although variable in length (depicted with the white dashed lines), each of the human (h) MICAL protein family members contains the same core domains as Drosophila (d) Mical including a flavoprotein monooxygenase (FM) domain (also called the redox or MO domain), a single calponin homology domain, and a single LIM domain. Multiple different splice forms of the MICALs have also been identified – some without the C terminus – as detailed in a recent review^67^. (**b**) Amino acid sequence alignments show that each of the human MICALs (M1, M2, M3), similar to Drosophila Mical (dM), contains three sequence motifs that define them as flavoprotein monooxygenases^69,91,92^. Each MICAL contains an exact match with each of the 11 residues of the ADP binding region of FAD binding proteins (GxGxxG motif^91^), where (+) indicates that MICALs match the consensus, (*) indicates that MICALs match the highly important conserved residues, and (.) indicates the conserved spacing of these residues within these motifs. The MICALs also have well-conserved FAD Fingerprint 2 (GD) and DG motifs, which are additional distinguishing features of flavoprotein monooxygenases^69,92^. Note, however, that MICAL-1 has a “naturally” occurring substitution of an alanine residue instead of the important aspartate residue in the DG motif. light blue = hydrophobic and cysteine residues, purple = acids, green = bases, yellow = proline, and orange = glycine. The proline (*) in the FAD fingerprint 2 is also likely to be conserved. (**c**–**f**) Each of the human MICALs binds FAD. The isoalloxazine ring system within flavins generates the yellow/orange color of FAD and FMN and is also responsible for light absorption in the UV and visible spectral range such that the oxidized form of FAD has two peaks at ~360 nm and ~450 nm^93^. Likewise, each of the purified human MICAL proteins, similar to Drosophila Mical^76^, has a UV-visible light absorption spectra with peaks at ~360 nm and ~450 nm (black lines) – and denaturation of the MICAL proteins releases FAD, which underlies this absorption spectra (green line). Note also that the flavin is shielded to some extent from absorbing light by the protein backbone of each of the MICAL proteins (e.g., compare the absorbance levels and wavelength of the black and green lines). [MICAL] = 20 μM. (**g**,**h**) Each of the human MICALs consumes the co-enzyme NADPH (**g**), preferring it over the related pyridine nucleotide coenzyme NADH (**h**), as observed by measuring the change in absorbance at 340 nm (NADPH and NADH absorb light at 340 nm, while the products of the conversion/consumption, NADP^+^ and NAD^+^, do not). Buffer only is the buffer used to store the MICAL proteins. [MICAL] = 600 nM, [NADPH] = 100 μM, [NADH] = 100 μM.

Mical is the sole Drosophila member of the MICAL family of proteins, which also includes three human protein family members coded for by three separate genes, *MICAL-1*, *MICAL-2*, and *MICAL-3* (Fig. 1a^12,18^). Each member of the MICAL family of proteins contains a similar protein organization, and includes a redox enzymatic domain, a calponin homology (CH) domain, a LIM domain, and a number of Src-homology 3 (SH3)-domain (PxxP) binding motifs (Fig. 1a^12,18^). At the C-terminus, MICAL family proteins also contain a region that interacts with the Plexin transmembrane receptor^12,18,19^, which is a receptor for one of the largest families of extracellular guidance cues, the Semaphorins^20,21^. The MICAL family of proteins are ubiquitously expressed (Reviewed in^18,22–25^) and they have been found to regulate multiple cellular events in different tissues including cell morphology and positioning^11,13–15,26–30^, axon growth/guidance^12,14,19,31,32^, synaptogenesis/neuronal plasticity^14,33,34^, dendritic arborization^35^, muscle formation^14,33^, cardiovascular function^29,32^, cell division/cytokinesis^36,37^, exocytosis^38,39^, and cell viability^40^. Likewise, altered levels of MICAL expression and polymorphisms in MICAL have been linked with different neuronal and non-neuronal pathologies including cancer^17,32,41–47^, diabetic nephropathy^30^, blood brain barrier dysfunction^29^, muscular dystrophy^48^, liver disease^49^, infectious susceptibility^50,51^, epilepsy^52^, neurological disorders^53–57^, neurodegenerative disease^58^, aging^59^, skeletal anomalies^60^, and obesity^61^. Yet, Drosophila Mical remains the best-characterized MICAL family member, and the actin regulatory properties of three mammalian members of the MICAL family of proteins, including comparisons among the different family members, remain poorly defined.

Herein, we find that each of the human MICAL proteins is an F-actin disassembly enzyme. Our results reveal that human MICALs-1, 2, and 3 directly associate with actin filaments, which activate the MICALs to catalyze enzyme reactions that selectively oxidize actin. This MICAL-mediated oxidation of actin, dismantles filaments, inhibits polymerization, is counteracted by SelR/MsrB reductases, and regulates cellular remodeling *in vivo*. Interestingly, differences among the catalytic and F-actin regulatory activities of the MICALs also exist, including that MICAL-1, which is the most divergent of the MICALs, has a single naturally-occurring amino acid substitution that allows it to have higher basal enzymatic activity and consume NADPH more robustly in the absence of its F-actin substrate than the other MICALs. Collectively, our results define the MICALs as an important new phylogenetically-conserved family of redox-acting actin filament disassembly factors.

## Results

### MICAL Family Proteins are Redox Enzymes

Both Drosophila Mical and mammalian MICALs form the MICAL family of large, multidomain cytosolic proteins (Fig. 1a), which direct multiple different cellular behaviors and physiological functions (e.g.^11–17,19,26–33,35–38,40,43^). We have recently identified Drosophila Mical as an actin filament disassembly factor^11,13,14^ and work using purified mammalian MICAL proteins has determined that at least some of the mammalian MICALs also exhibit direct effects on actin stability (e.g.^15,32,37,62^). However, since an in-depth characterization and comparison of the different MICAL family members and their mechanisms of action is lacking, we set out to define each MICAL family member and its effects on F-actin.

Our previous results revealed that the oxidoreductase (redox) region of Drosophila Mical (Fig. 1a) is sufficient to post-translationally oxidize and disassemble actin filaments^11,13,14^, but Mical’s calponin homology (CH) domain (Fig. 1a) may also assist the redox region in its catalytic efficiency^63^. Therefore, so that we could compare and contrast the actin regulatory properties of each of the MICAL family members (and do it in the same species), we expressed and purified recombinant proteins containing the redox and CH portions of each of the three human MICALs, hMICAL^redoxCH^-1, hMICAL^redoxCH^-2, and hMICAL^redoxCH^-3 (Supplementary Figures 1–3). The redox regions of both invertebrate and vertebrate members of the MICAL family of proteins are highly conserved with as high as 63% identity between Drosophila and human family members (Supplementary Figure 4a). Likewise, primary sequence analysis of Drosophila and human family members revealed that each of them has within their redox enzymatic domain three well-conserved amino acid motifs that are found in proteins that bind and utilize the redox enzyme co-factor flavin adenine dinucleotide (FAD) (Fig. 1a,b^12^). Experimental analysis also identified that purified human MICAL-1^redoxCH^, MICAL-2^redoxCH^, and MICAL-3^redoxCH^ proteins, like Drosophila Mical^redoxCH^, are yellow in color, a distinguishing feature of flavin binding proteins (flavoproteins), and also exhibited an absorption spectra similar to flavoproteins (Fig. 1c–f, Supplementary Figures 1–3). Protein denaturation studies also supported these observations, and demonstrated that recombinant Drosophila Mical and all three human MICAL proteins bind the flavin FAD non-covalently in a 1:1 MICAL:FAD stoichiometry (Supplementary Figure 4b,c). Thus, each of the human MICALs, like Drosophila Mical, is an FAD binding protein.

Proteins that bind FAD typically perform oxidation-reduction reactions, so we wondered if all members of the MICAL family of proteins do the same. Primary sequence analyses revealed that MICAL family proteins are similar to a class of redox enzymes called flavoprotein monooxygenases^12^, and this similarity was also observed when the redox portion of mouse MICAL-1 was crystallized, examined structurally, and found to be most similar to the flavoprotein monooxygenase enzyme *p*-hydroxybenzoate hydroxylase (pHBH)^63–65^. Flavoprotein monooxygenases such as pHBH utilize the pyridine nucleotide coenzyme nicotinamide adenine dinucleotide phosphate (NADPH) in their redox reactions^18,66^, so we compared the redox properties of each member of the human MICAL family. Our results revealed that each mammalian MICAL family member, like Drosophila Mical, consumes NADPH (Fig. 1g). Furthermore, we found that similar to Drosophila Mical, hMICAL-1, 2, and 3 are more active in the presence of NADPH than with the related pyridine nucleotide coenzyme NADH (Fig. 1h). However, we also noticed differences in the redox properties among the different MICAL family members. For example, hMICAL^redoxCH^-1 is substantially more active than the other members of the MICAL family in the absence of a substrate and consumes high levels of NADPH on its own (Fig. 1g). In contrast, hMICAL^redoxCH^-2, hMICAL^redoxCH^-3, and Drosophila MICAL^redoxCH^ are most similar to each other with regards to NADPH consumption (Fig. 1g), and most similar to PHBH, in that they each have relatively weak NADPH consumption activity on their own (i.e., in the absence of a substrate). Collectively, these results indicate that all members of MICAL family of proteins exhibit redox activity – although there are notable differences between family members in their catalytic properties.

### Each Human MICAL Family Member Directly Binds and Dismantles Actin Filaments

**Figure 2:**
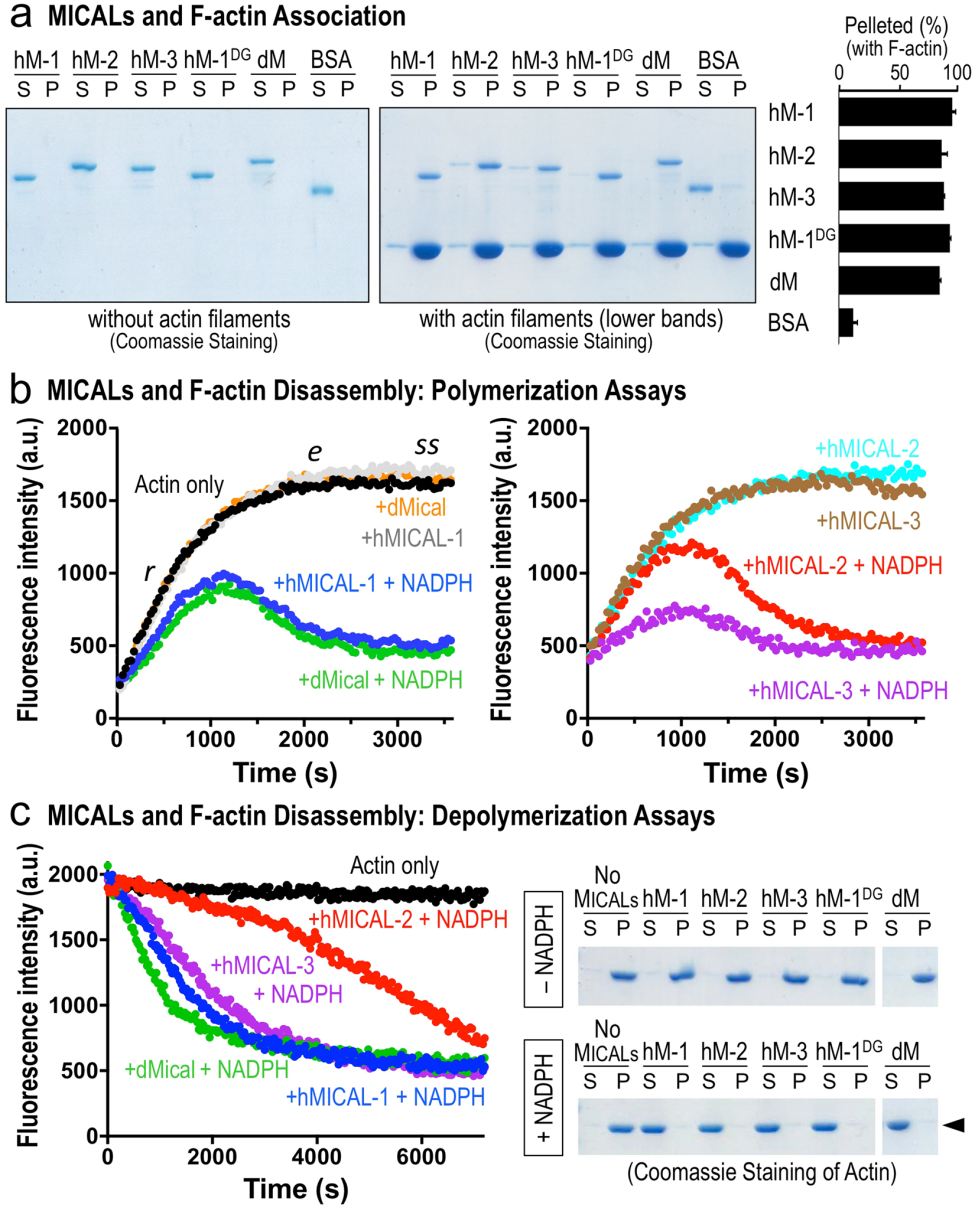
Each human MICAL family member directly binds and disassembles actin filaments. (**a**) Each of the purified human MICAL^redoxCH^ proteins, hMICAL-1 (hM-1), hMICAL-2 (hM-2), hMICAL-3 (hM-3), and hMICAL-1 DG (hM-1^DG^), like Drosophila Mical^redoxCH^ (dM), associates with F-actin as revealed by actin co-sedimentation/pelleting assays in which Coomassie blue stained gels are shown. Notice that after high-speed centrifugation, each of the purified MICALs is present in the soluble (S) fraction (left gel). In the presence of purified actin filaments, however, each of the MICALs is present in the pellet (P) fraction (right gel). The percentage (±the standard error of the mean (SEM)) of different MICAL proteins in the pelleted fraction following incubation with purified F-actin was quantified by densitometry (*n* = 3). Bovine serum albumin (BSA) was used as a control and was predominantly found in the S fraction. [MICALs] = 600 nM, [BSA] = 600 nM, [actin] = 2.3 μM. (**b,c**) Each of the human MICALs alters actin polymerization and depolymerization. Pyrene-labeled actin was used to monitor both the polymerization and depolymerization of actin using standard approaches, where the fluorescence intensity (a.u. (arbitrary units)) of the pyrene-labeled actin polymer is substantially higher than the pyrene-labeled actin monomer. Each of the MICALs and the reaction conditions are color-coded here and in Figs 3,5. (**b**) Standard pyrene-actin polymerization assay. No filaments are present at Time = 0, when MICALs are added to actin and polymerization is induced. As can be observed by following the characteristic increase in fluorescence intensity over time, the addition of each of the purified human MICALs (in the presence of their co-enzyme NADPH) decreases the rate (*r*), extent (*e*), and steady-state level (*ss*) of actin polymerization (as compared to an actin only control, black dots; or MICAL only (no NADPH) + actin controls; or NADPH only + actin controls (not shown; see^11^)). Notably, MICAL (NADPH) induces actin polymerization to slow-down over time, which is followed by a substantial decrease in the extent of polymerization, the rapid depolymerization of F-actin, and the inability of actin to reinitiate polymer formation. Thus, because each of the MICALs acts on F-actin^11,14^ (and herein), our data in the polymerization assays are consistent with a model in which as the actin begins to polymerize, the MICALs oxidize individual subunits of the polymer, which disassembles the polymerizing actin. This slows polymerization – and also creates fewer and fewer unoxidized monomers that can be used for polymerization. [MICAL-1] = 300 nM, [dMical and MICALs-2, 3] = 600 nM, [NADPH] = 100 μM, [Actin] = 1.15 μM. (**c**) Purified human MICALs+/– NADPH was added to actin that was polymerized and kept in buffer conditions that favored polymerization (as can be seen with the steady-state fluorescence intensity level of the actin only control (black dots); or actin with each of the MICALs without NADPH (not shown; see^11^)). The addition of each of the human MICALs (in the presence of NADPH) induces actin depolymerization (decreasing fluorescence intensity that can be followed over time in the pyrene actin depolymerization assay (left)). [MICAL-1] = 300 nM, [dMical and MICALs-2, 3] = 600 nM, [NADPH] = 100 μM, [Actin] = 1.15 μM. The extent of this MICAL (NADPH)-dependent actin depolymerization is also observed when MICAL-treated F-actin was subjected to high-speed centrifugation to differentiate F-actin (P) from G-actin (S). As can be observed in these Coomassie stained gels, each of the human MICALs (in the presence of NADPH) substantially increases the ratio of G-actin to F-actin (arrowhead). [MICAL-1] = 300 nM, [dMical and MICALs-2, 3] = 600 nM, [NADPH] = 100 μM, [Actin] = 1.15 μM. Unprocessed original scans of gels are shown in Supplementary Fig. 11.

Drosophila Mical directly binds and disassembles F-actin in an NADPH-dependent manner^11,13^, so we wondered if each of the human MICAL proteins also functions in a similar way. We first looked at the ability of hMICAL^redoxCH^-1, hMICAL^redoxCH^-2, and hMICAL^redoxCH^-3 proteins to associate with actin filaments using actin co-sedimentation assays. Like Drosophila Mical, co-sedimentation assays revealed that all three human MICALs physically associate with F-actin (Fig. 2a). Likewise, we found that these MICAL–F-actin interactions are specific, since similar to Drosophila Mical, human MICALs did not directly associate with microtubules (Supplementary Figure 5a). Thus, we wondered if human MICALs affect F-actin stability. Using different actin polymerization and depolymerization assays including pyrene-actin and sedimentation analyses, we found that each of the human MICALs, similar to Drosophila Mical^11^, altered actin polymerization and induced the disassembly of F-actin (Fig. 2b,c). Namely, when added prior to actin polymerization, each of the MICALs induces actin polymerization to slow-down over time, which is followed by a substantial decrease in the extent of polymerization, the rapid depolymerization of F-actin, and the inability of actin to reinitiate polymer formation (Fig. 2b^11^). In a similar way, when added to polymerized actin, each of the MICALs induces the disassembly of filaments (Fig. 2b^11^). Moreover, like Drosophila Mical^11^, these effects on polymerization and depolymerization were specific to F-actin, as microtubule polymerization and depolymerization dynamics were not affected by human MICALs (Supplementary Figure 5b). Further analysis revealed that each of the human MICALs effects on F-actin, like Drosophila Mical^11^, was more robust in the presence of NADPH versus NADH (Supplementary Figure 6a,b). These results therefore reveal that each of the human MICALs directly associates with actin filaments – inducing both F-actin disassembly and preventing normal F-actin reassembly.

### Actin Filaments Serve as a Direct Substrate for Each MICAL Family Member

**Figure 3:**
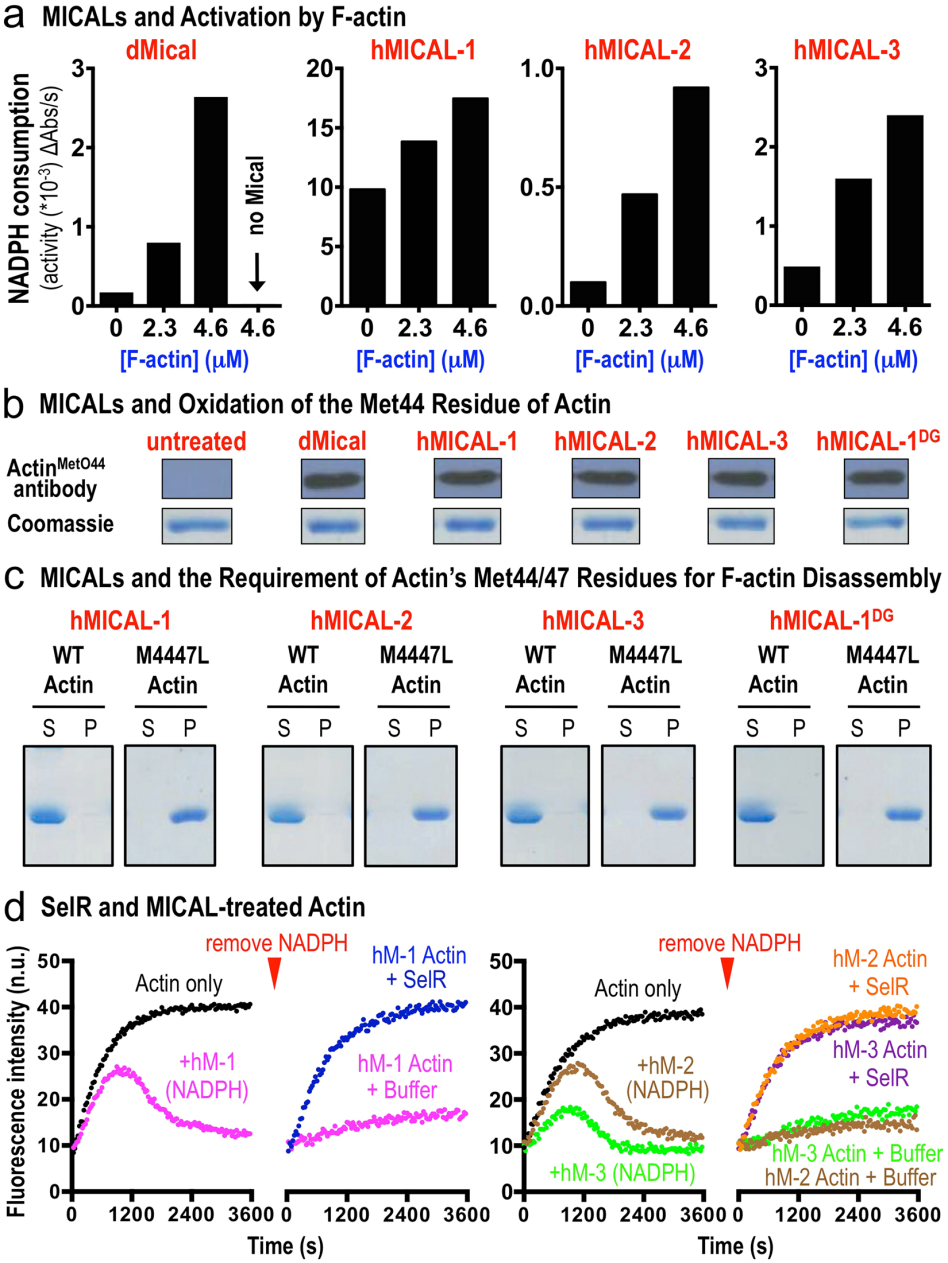
Each human MICAL family member is activated by actin filaments, oxidizing the Met44 residue of actin to induce F-actin disassembly. (**a**) The enzymatic activity of each of the human (h) MICALs, similar to Drosophila (d) Mical, notably increases in the presence of F-actin. Enzyme activity was determined by consumption (conversion of NADPH to NADP^+^) of MICAL’s co-enzyme NADPH, which was monitored by recording the light absorbance at 340 nm wavelength/time. [MICALs] = 600 nM; [NADPH] = 200 μM. (**b**) Each of the human MICALs oxidizes the Met44 residue of actin, as determined using an antibody that specifically recognizes Mical-oxidized actin (actin^MetO44^ ^16^). Similar amounts of actin (lower panel) are present in all experiments. Note, as described in the methods, actin was polymerized to generate F-actin and 600 nM of each of the human MICALs and 200–300 µM of NADPH were added to 1.15 μM of F-actin at room temperature for 2 hours. (**c**) Each of the human MICALs requires actin’s Met44 and Met47 residues to disassemble F-actin. Drosophila Mical oxidizes the Met44 and Met47 residues of actin^13^ and so we substituted as previously described a chemically related leucine residue for the methionine 44 and 47 residues in actin (M4447L)^13^ to determine if each of the human MICALs also requires these residues to disassemble F-actin. Sedimentation assays reveal that each of the human MICALs robustly disassembles filaments composed of wild-type (WT) actin but not M4447L actin. [Actin] = 1.15 μM; [MICALs-1, 1^DG^, 2, and 3] = 600 nM; [NADPH] = 200–300 μM. (**d**) The stereospecific methionine sulfoxide reductase SelR/MsrB restores the polymerization properties of actin treated with each of the human MICALs. Pyrene-actin assays, where the fluorescence is higher in the polymerized state, reveal that SelR restores the polymerization of human MICAL-1 (hM-1), human MICAL-2 (hM-2), and human MICAL-3 (hM-3) treated actin. Buffer (buffer that SelR is stored in), n.u. (normalized units between the 2 graphs). [MICAL-1] = 100 nM, [dMical and MICALs-2, 3] = 600 nM, [NADPH] = 100 μM, [Actin] = 1.15 μM. Unprocessed original scans of gels/blots are shown in Supplementary Fig. 11.

We next sought to examine the mechanism by which the human MICALs alter actin dynamics. Our previous results revealed that actin filaments exhibit the characteristics of a direct Mical substrate, robustly increasing the enzyme activity of Drosophila Mical (Fig. 3a^11^). Likewise, we found that the enzyme activity of each of the human MICALs, as judged by an increase in the consumption of the co-enzyme NADPH, increased in the presence of actin filaments (Fig. 3a). We also had previously determined that the methionine (Met) 44 and 47 residues of actin are the sites of Drosophila Mical post-translational oxidation of actin^13^, and that mutations to those residues makes actin resistant to Drosophila Mical^13^. Likewise, using an antibody that specifically recognizes the Mical-oxidized Met44 residue of actin^16^, we found that Met44 is oxidized by human MICALs 1, 2, and 3 (Fig. 3b) – and that mutating the Met44 and Met47 residues of actin prevents each of the human MICALs from disassembling F-actin (Fig. 3c). Moreover, we previously determined that the methionine sulfoxide reductase enzyme SelR/MsrB specifically reverses Drosophila Mical-mediated oxidation of actin through reduction of the oxidized Met44 and Met47 residues of actin^14,16^ – and that this SelR/MsrB-mediated effect also restores the polymerization properties of Mical-treated actin^14,15^. Thus, we wondered if each member of the MICAL family also works in a similar way to oxidize actin and if SelR/MsrB could reverse this modification and restore the polymerization properties of human MICAL-treated actin. Indeed, we found that the methionine sulfoxide reductase enzyme SelR/MsrB restored the polymerization properties of actin that had been treated with each of the human MICALs (Fig. 3d). These results therefore reveal that actin serves as a direct substrate of mammalian MICALs and that the Met44 and 47 residues are essential for the ability of each of the human MICALs to disassemble F-actin. Likewise, since SelR/MsrB enzymes specifically catalyze the reduction of the *R* isomer of methionine sulfoxide (methionine-*R*-sulfoxide) to methionine (reviewed in^18,25^), these results also indicate that each MICAL family member oxidizes actin stereospecifically in the *R*-isomer to generate actin Met44,47-*R*-sulfoxide (actin^Met(*R*)O–44,47^ ).

### MICAL-1 exhibits notable differences in its activation and catalytic activity in comparison to other MICAL family members

**Figure 4:**
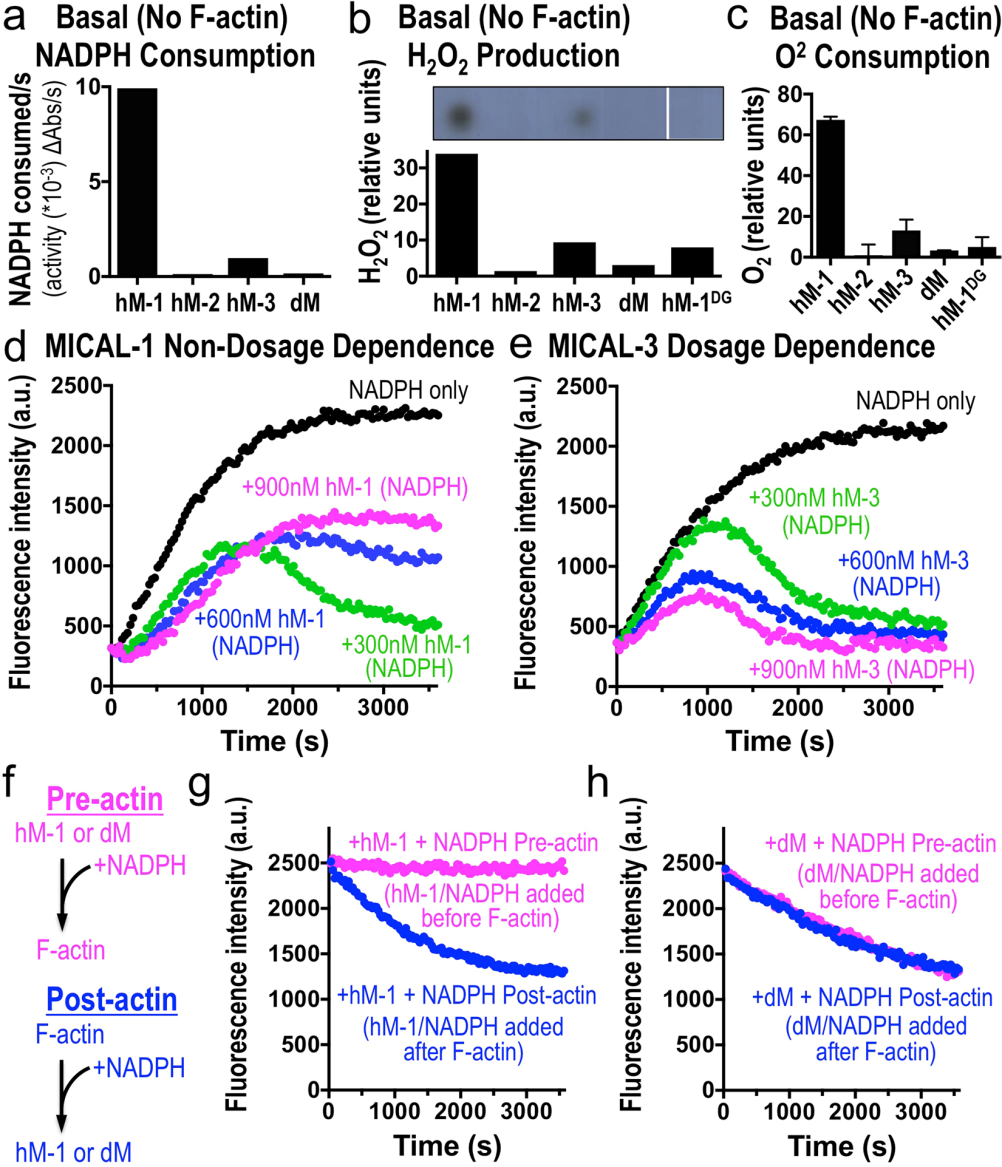
MICAL-1 exhibits notable activation and catalytic activity differences in comparison to other MICALs. (**a**) The enzyme activity of human MICAL-1 (hM-1) in the absence of its substrate F-actin is substantially higher than that of the other MICALs, as judged by consumption of NADPH. In particular, basal (in the *absence* of F-actin) NADPH consumption by MICAL-1 is close to 100 times more NADPH consumption *per second* than hMICAL-2, 60 times more NADPH consumption *per second* than Drosophila Mical, and over 10 times more NADPH consumption *per second* than hMICAL-3. [MICALs] = 600 nM, [NADPH] = 200 μM. (**b**) hM-1 generates substantially more hydrogen peroxide (H_2_O_2_) than other MICALs (all assays done in the presence of NADPH and the absence of F-actin). Upper panel, chemiluminescent detection of H_2_O_2_ produced by MICALs, reveals significant H_2_O_2_ production by hM-1 in comparison to others MICALs that is quantified using a different assay, a bioluminescent assay (lower panel). Also note in both panels that hM-1^DG^ generates substantially less H_2_O_2_ than hM-1. [MICALs] = 600 nM; [NADPH] = 100 μM. (**c**) hM-1 consumes substantially more oxygen (O_2_) than other MICALs (all assays done in the presence of NADPH and the absence of F-actin). Also note that hM-1^DG^ consumes substantially less O_2_ than hM-1. [MICALs] = 600 nM; [NADPH] = 200 μM. (**d**,**e**) Pyrene-actin assays reveal that hMICAL-1 (**d**), unlike hMICAL-2 (Supplementary Figure 7d), hMICAL-3 (**e**) and Drosophila Mical^11^, exhibits decreasing effects on F-actin when higher levels of it are added to F-actin. These effects are consistent with hMICAL-1 exhibiting such rapid consumption of NADPH in the absence of its F-actin substrate that NADPH becomes limiting in allowing MICAL-1 to alter F-actin dynamics (see also main text). [Actin] = 1.15 μM, [NADPH] = 100 μM. (**f**–**h**) hMICAL-1 generates a high-level of basal H_2_O_2_, but does not use H_2_O_2_ to modify actin dynamics. (**f**) To test if MICALs use H_2_O_2_ to disassemble F-actin we made use of MICAL-1’s high level of basal activity to produce H_2_O_2_ in the presence of NADPH, and added NADPH either before (pre) or after (post) the addition of F-actin. In short, we reasoned that if H_2_O_2_ (or another stable oxidant) was being used by MICALs to modify actin, it would not matter in which order we added F-actin to the same tube. However, if H_2_O_2_ (or another stable oxidant) was not being used to modify F-actin, MICAL-1 would exhaust its supply of NADPH generating H_2_O_2_ prior to the addition of F-actin, and would therefore have decreased effects on F-actin dynamics. (**g**) MICAL-1 no longer disassembles F-actin when it is incubated with NADPH prior to the addition of F-actin (pink), revealing it does not use H_2_O_2_ or another stable/generally-released oxidant to disassemble F-actin. (**h**) As a control, Drosophila Mical, which has a low rate of basal NADPH consumption (and thus retains NADPH when its F-actin substrate is not present), exhibits similar disassembly of F-actin in both the Pre- and Post- incubation conditions. [Actin] = 1.15 μM, [dMical and MICAL-1] = 600 nM, [NADPH] = 100 μM. Unprocessed original scans of blots are shown in Supplementary Fig. 11.

Our results herein reveal that each member of the MICAL family of proteins uses its enzyme activity to covalently modify and disassemble F-actin, but we also noticed that MICAL-1 exhibited pronounced differences in this catalytic activity when compared to other MICAL family members. In particular, in the absence of their F-actin substrate, Drosophila Mical and human MICALs 2 and 3 consumed relatively low amounts of their essential co-enzyme NADPH (Figs 1g, 3a, 4a^13^). MICAL-1, in contrast, consumed high levels of NADPH in the absence of its F-actin substrate (Figs 1g, 3a, 4a), such that it basally (in the *absence* of F-actin) consumed close to 100 times more NADPH per second than hMICAL-2 and 60 times more NADPH per second than Drosophila Mical (Fig. 4a). Thus, MICAL-1, in contrast to other MICAL family members, is highly active in the absence of its substrate. In the class of enzymes that includes pHBH and MICALs, consumption of NADPH in the absence of a substrate leads to the production of hydrogen peroxide (H_2_O_2_) – since no substrates are present to directly oxidize (Supplementary Figure 7a^18,67^). Likewise, using several different assays to monitor H_2_O_2_ production in the absence of a substrate, we found that hMICAL-1 produced higher levels of H_2_O_2_ than other MICAL family members (Fig. 4b, Supplementary Figure 7b). Furthermore, using an assay to monitor the usage of oxygen, we found that MICAL-1 consumed much higher levels of oxygen in the absence of a substrate in comparison to other MICALs (Fig. 4c). Thus, MICAL-1 shows marked differences in its catalytic properties from other MICALs, including a robust enzymatic activity in the *absence* of its F-actin substrate.

To further investigate these differences among the MICALs we wondered what effect this MICAL-1-mediated large increase in H_2_O_2_ production might have on F-actin dynamics. It has been suggested that the MICALs use H_2_O_2_ to exert their effects^62,68^. However, our previous results using multiple different approaches and those of others demonstrated that MICALs do not use the non-specific release of H_2_O_2_ or other diffusible oxidants to alter F-actin dynamics^11,13,14,18,37^, but need to be in close proximity to F-actin, which directly activates Mical to oxidize the Met44 and Met47 residues of actin^13^. Likewise, our results presented herein also support these conclusions for each of the mammalian MICALs, since we observe an excessive difference in H_2_O_2_ production between human MICAL-1 and the other MICALs (Fig. 4b, Supplementary Figure 7b), but not a corresponding increase in MICAL-1’s effects on F-actin dynamics (Fig. 2b,c). Similarly, we found that raising the levels of MICAL-1 (from 300 nM to 900 nM) increased H_2_O_2_ production but decreased F-actin alterations (Fig. 4d, Supplementary Figure 7c), further arguing against a role for the diffusible release of H_2_O_2_ or other oxidants in MICAL-mediated F-actin alterations. Indeed, since hMICAL-2, hMICAL-3 and Drosophila Mical, which have a low basal NADPH consumption rate, increased their effects on F-actin dynamics in a concentration-dependent manner (Fig. 4e, Supplementary Figure 7d^11^), our results argue that MICAL-1 exhibits such rapid consumption of NADPH in the absence of its F-actin substrate that NADPH (and not H_2_O_2_ [which is present in high quantities in the MICAL-1 reaction; Fig. 4b, Supplementary Figure 7b]) becomes limiting in allowing MICAL-1 to modify F-actin. Thus, our results indicate that the release of H_2_O_2_ plays no role in MICAL-1 mediated F-actin disassembly.

To further test the role of H_2_O_2_ in MICAL-mediated F-actin disassembly, we made use of MICAL-1’s high level of basal activity and designed one additional experiment in which we incubated MICAL-1 with NADPH either before (pre) or after (post) the addition of F-actin (Fig. 4f). We reasoned that if H_2_O_2_ is the means by which MICAL-1 induces F-actin disassembly, there would be no difference in F-actin disassembly between these conditions. However, our results revealed that MICAL-1 no longer disassembles F-actin when it is incubated with NADPH prior to the addition of F-actin (Fig. 4g) – supporting that even though MICAL-1 produces high levels of H_2_O_2_ in this pre-reaction condition (i.e., without its F-actin substrate [Fig. 4f]), H_2_O_2_ has no effect on F-actin disassembly (Fig. 4g). MICAL-1, therefore, because of its high-rate of basal activity uses-up NADPH prior to the addition of F-actin, such that it can no longer use NADPH in its reaction to modify actin. Drosophila Mical, in contrast, which has a low rate of basal NADPH consumption (Figs 1g, 3a and 4a), retains NADPH levels in the absence of its F-actin substrate, and exhibited similar effects on F-actin disassembly in each condition (Fig. 4h). Indeed, when we added more NADPH into the MICAL-1 pre-reaction condition tube (Supplementary Figure 7e), MICAL-1 now proceeded to induce F-actin disassembly (Supplementary Figure 7f). Thus, the general release of H_2_O_2_ is not the means by which MICAL-1 or any of the MICALs post-translationally oxidize and regulate F-actin disassembly. These results therefore further support our previous results with Drosophila Mical^11,13,14^ and those above that the MICALs need to be in close proximity to F-actin, which directly activates the MICALs to oxidize the Met44 and 47 residues of actin.

### MICAL-1 exhibits a single amino acid alteration that produces high levels of catalytic activity in the absence of its F-actin substrate

**Figure 5:**
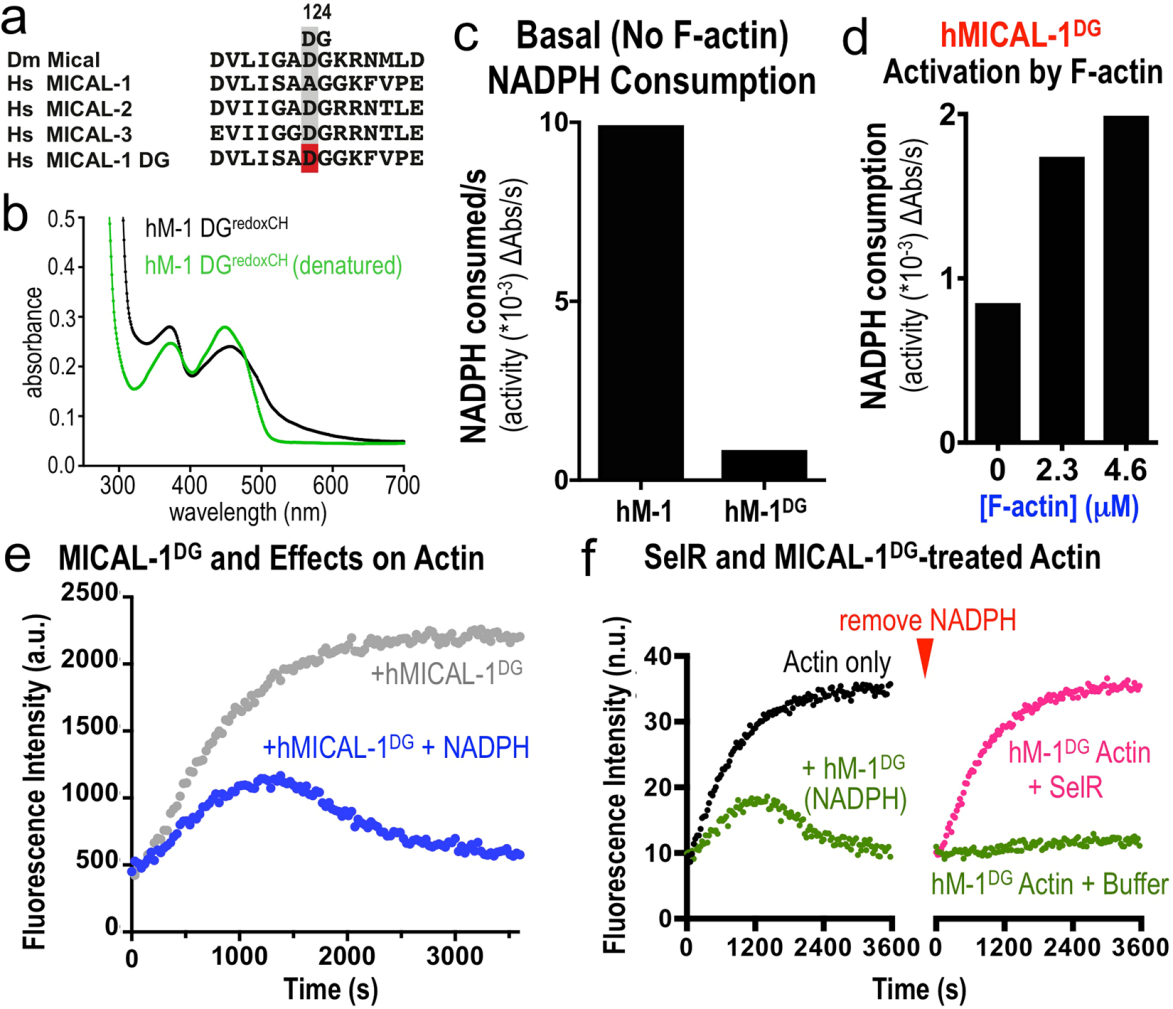
MICAL-1 exhibits a single amino acid alteration in its DG motif that produces high levels of catalytic activity in the absence of its F-actin substrate. (**a**) MICAL-1 has a “naturally” occurring substitution of an alanine (A) residue instead of the important aspartate (D) residue in the DG (Conserved) motif that is present in the other MICAL family members. Using site-directed mutagenesis we converted the alanine residue within the DG motif of MICAL-1 to an aspartate residue and thereby generated a MICAL-1 protein similar to the other MICAL family members (MICAL-1^DG^). (**b**) Purified MICAL-1^DG^ protein still exhibits the hallmarks of an FAD-binding protein. In particular, MICAL-1^DG^ maintains its UV-visible light absorption spectra (with peaks at ~360 nm and ~450 nm, black lines), and denaturation of the MICAL-1^DG^ releases FAD, which underlies this absorption spectra (green line). [MICAL-1^DG^] = 20 μM. (**c**) Converting the alanine residue within the DG motif of MICAL-1 to an aspartate residue (MICAL-1^DG^), substantially reduces (12-fold *per second*) the basal (in the absence of its F-actin substrate) enzyme activity of MICAL-1, as judged by the consumption of NADPH. [MICALs] = 600 nM, [NADPH] = 200 μM. (**d**) The enzymatic activity of MICAL-1^DG^, similar to other MICALs, is notably increased in the presence of F-actin. Enzyme activity was determined by the consumption (conversion of NADPH to NADP^+^) of MICAL’s co-enzyme NADPH, which was monitored by recording the light absorbance at 340 nm wavelength/time. [MICAL-1^DG^] = 600 nM, [NADPH] = 200 μM. (**e**) MICAL-1^DG^, similar to unaltered MICAL-1 and other MICAL family members, induces actin polymerization to slow-down over time, which is followed by a substantial decrease in the extent of polymerization, the rapid depolymerization of F-actin, and the inability of actin to reinitiate polymer formation. [Actin] = 1.15 μM, [MICAL-1^DG^] = 600 nM, [NADPH] = 100 μM. Here, as in Fig. 2b, pyrene-labeled actin was used to monitor both the polymerization and depolymerization of actin using standard approaches, where the fluorescence intensity (a.u. (arbitrary units)) of the pyrene-labeled actin polymer is substantially higher than the pyrene-labeled actin monomer. (**f**) Similar to unaltered MICAL-1 and other MICAL family members, the stereospecific methionine sulfoxide reductase SelR/MsrB restores the polymerization properties of actin treated with MICAL-1^DG^. See also Fig. 3d. Buffer (buffer that SelR is stored in), n.u. (normalized units between the 2 graphs). [Actin] = 1.15 μM, [MICAL-1^DG^] = 600 nM, [NADPH] = 100 μM.

Since MICAL-1, unlike other MICAL family members (and MICAL-class enzymes like pHBH), has a high level of enzymatic activity in the absence of a substrate, we wondered what molecular variation(s) might be underlying this difference. Interestingly, comparing the sequences for the different MICALs revealed that the critical aspartate (D) amino acid residue in the DG motif of the flavin adenine dinucleotide (FAD) binding motif was instead an alanine (A) residue in MICAL-1 (Fig. 1b). The DG motif (also called the Conserved Motif^69^;) is critical for binding the pyrophosphate moiety of FAD (reviewed in^67^), and so this “naturally-occurring” MICAL-1-specific substitution of a nonpolar alanine residue for the charged aspartate residue would be predicted to alter the positioning and flexibility of the FAD. This “naturally-occurring” MICAL-1-specific substitution might also enable MICAL-1 to be more catalytically active in the absence of its F-actin substrate than other MICALs. To test this hypothesis, we used site-directed mutagenesis to convert the alanine residue within the DG motif of MICAL-1 to an aspartate residue and thereby generated a MICAL-1 protein similar to the other MICAL family members (MICAL-1^DG^; Fig. 5a, Supplementary Figure 8). Our initial analysis of this MICAL-1^DG^ protein revealed that it was similar to wild-type MICAL-1 (and other MICAL family members) – yellow in color (Supplementary Figure 8) and displaying the enzymatic features and absorption spectra of MICAL-class enzymes (Fig. 5b; Supplementary Figure 4b,c). Yet, strikingly, we also found that making this DG substitution decreased MICAL-1’s basal consumption of NADPH by close to 12-fold (1200%) per second (Fig. 5c) – enabling MICAL-1^DG^ to consume much lower levels of oxygen and generate far less H_2_O_2_ than wild-type MICAL-1 (Fig. 4b,c). However, making this DG substitution did not decrease MICAL-1’s actin regulatory properties – since we observed that MICAL-1^DG^ still bound actin filaments (Fig. 2a), was activated by F-actin (Fig. 5d), oxidized actin’s Met44 residue (Fig. 3b), and had robust and specific effects on both actin polymerization and depolymerization (Figs 2c, 5e; Supplementary Figure 5a,b). Furthermore, these MICAL-1^DG^ effects on F-actin dynamics were dependent on the Met44 and Met47 residues of actin and could be counteracted by SelR (Figs 3c, 5f). Therefore, despite the large differences in H_2_O_2_ production between MICAL-1^DG^ and wild-type MICAL-1, we did not observe any widespread differences in their actin regulatory abilities (Figs 2c, 5e). Indeed, our results revealed that MICAL-1^DG^ became more efficient than MICAL-1 at altering actin dynamics (Supplementary Figure 9) – because it now had a lower basal level of NADPH consumption and generated less H_2_O_2_. Thus, these results further support that the MICALs do not use general H_2_O_2_ production to exert their effects on actin dynamics. Furthermore, these results also indicate that the aspartate (D) to alanine (A) substitution that occurs “naturally” in MICAL-1 underlie differences in basal activity and H_2_O_2_ production between MICAL-1 and the other MICAL family members.

### Mammalian MICALs Regulate F-actin Disassembly and Cellular Remodeling ***In Vivo***

**Figure 6:**
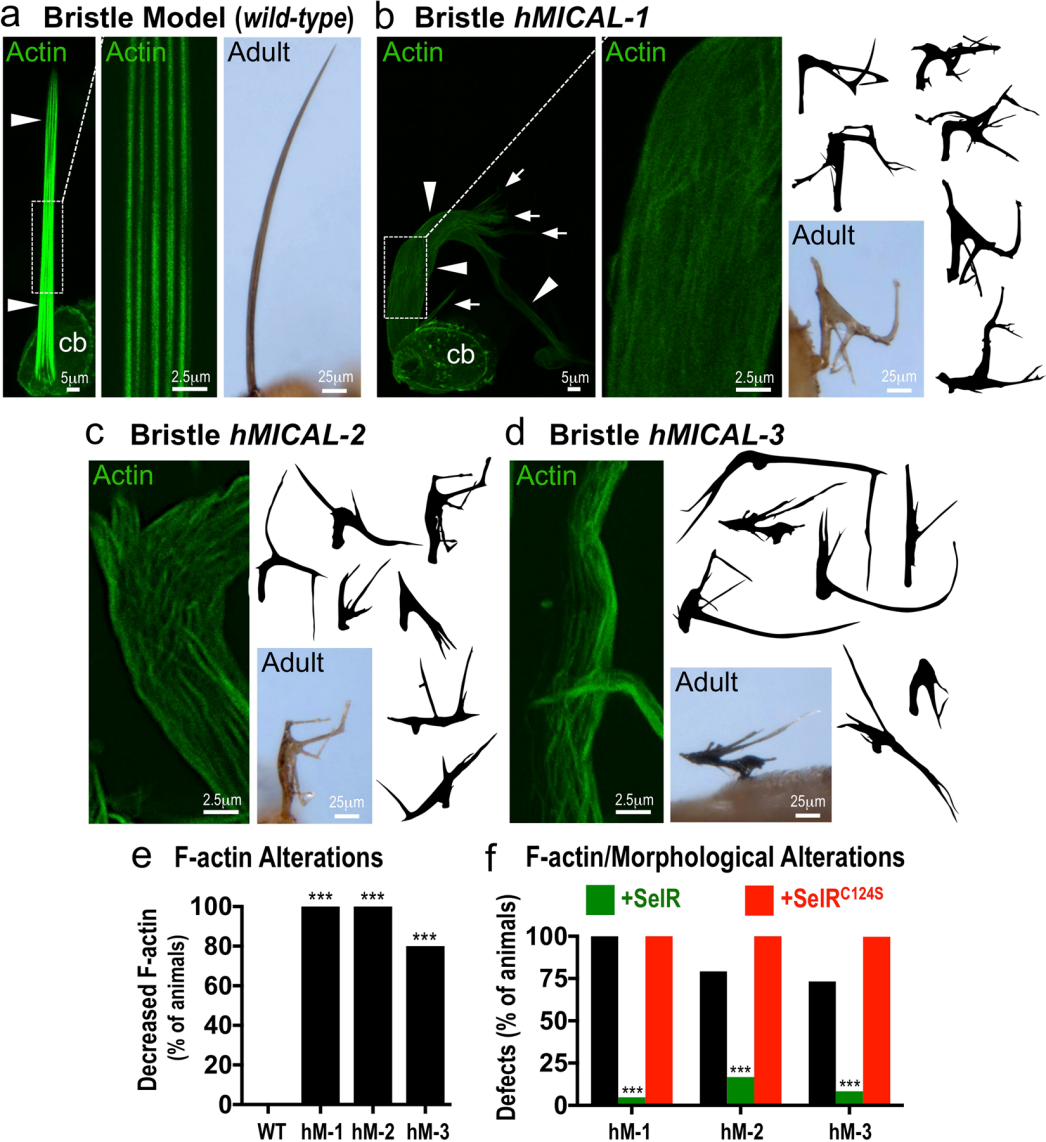
Each human MICAL family member generates F-actin disassembly and cellular remodeling *in vivo*. (**a**) Drosophila bristle cells serve as a model for examining actin organization and cellular morphology *in vivo*. Bristle cells are single cells composed of a cell body (cb) and a long F-actin-rich (green) cellular extension (arrowheads). The F-actin organization (green) in the bristle process can be easily observed throughout pupal development (left and the boxed region is shown at higher magnification in the middle), while the morphology of the single bristle cell can be observed both during development (left) and in adulthood (right). (**b**) Expression of *hMICAL-1*^*redoxCH*^ specifically in bristles generates alterations in F-actin organization and cellular morphology. Notice the dramatic alterations to the height, width, and shape of the bristles (arrowheads) including branches (arrows) that can be seen in developing pupae (left) and in the image and drawings of single bristles from adults (right). Notice also that a similar degree of F-actin (green) is present in patches around the periphery of the cell body (cb), but that the extent of F-actin (green) in the bristle process (arrowheads) in much less compared to the wild-type control in (**a**) – such that there is an absence of actin filaments and it is more difficult to discern F-actin/F-actin bundles. Also, note that the F-actin is no longer arrayed in linear projecting bundles (as in a), but in small filaments and bundles of filaments with no apparent organization (left and in the boxed region shown at higher magnification). (**c,d**) Similar dramatic alterations in F-actin organization and bristle morphology as observed with *hMICAL-1*^*redoxCH*^ (**b**) were also seen when *hMICAL-2*^*redoxCH*^ (**c**) and *hMICAL-3*^*redoxCH*^ (**d**) were specifically expressed in bristles. (**e**) Quantification of F-actin alterations following bristle expression of different human MICALs, as judged by animals that had a loss of parallel-arranged F-actin bundles. n = > 10 animals/genotype. (**f**) Quantification of F-actin/morphological defects present following bristle expression of different human MICALs, or in combination with SelR or a reductase-dead form of SelR (SelR^C124S^)^14^. Note that SelR significantly rescues the F-actin/morphological alterations induced by each of the human MICALs, while SelR^C124S^ does not rescue these defects (and enhances the defects induced by *hMICAL-2*^*redoxCH*^ and *hMICAL-3*^*redoxCH*^*). hM-1 (hMICAL-1*^*redoxCH*^*), hM-2 (hMICAL-2*^*redoxCH*^*), hM-3 (hMICAL-3*^*redoxCH*^*)*. Chi-Square Test; ***P < 0.0001; n = > 10 animals/genotype.

Our biochemical experiments reveal that each member of the MICAL family of proteins functions to directly induce actin disassembly. We conducted these experiments using purified proteins *in vitro* so we wondered if each MICAL family member could also exert similar effects on F-actin organization *in vivo*. Bristle cells in the model organism Drosophila have long-provided a high-resolution single cell system for characterizing F-actin alterations *in vivo* (Fig. 6a^67,70,71^) – and our previous results revealed that Drosophila Mical induces F-actin disassembly in a redox-dependent manner to remodel these cells^11,13,14^. We therefore turned to this model system to determine if each member of the MICAL family has a similar ability – and tested this by generating transgenic flies expressing human MICALs corresponding to the exact protein construct we used *in vitro*, hMICAL-1^redoxCH^, hMICAL-2^redoxCH^, and hMICAL-3^redoxCH^. Notably, expressing each of the human MICALs in developing bristle cells using the bristle-specific driver (*B11-GAL4*) resulted in widespread and dramatic effects to actin organization and cellular morphology. The normally bundled and parallel-arranged F-actin (Fig. 6a, left and middle) that gives rise to a long slightly-curved bristle process (Fig. 6b, right) was significantly altered by expressing MICALs 1, 2 or 3 within them (Fig. 6b–f). Specifically, MICALs-1, 2, and 3 were each sufficient to decrease the presence of actin filaments in extending bristle processes (Fig. 6b–e, and compare to Fig. 6a) and resulted in substantial remodeling of bristle cells to generate numerous abnormal bends and branches (Fig. 6b–d). Furthermore, SelR, but not an enzyme dead version of SelR (SelR^C124S^), rescued the F-actin and morphological defects induced by MICALs-1, 2, and 3 (Fig. 6f), indicating that each of the human MICALs uses its redox activity to alter actin dynamics and cellular remodeling *in vivo*. Thus, as we observed *in vitro* using purified proteins, each of the human MICALs exerts specific negative effects on F-actin organization *in vivo* – effects that significantly remodel cells.

## Discussion

Dynamic assembly and disassembly of the actin cytoskeleton allows cells to perform the functions necessary for normal development and physiology. Yet, while we have a considerable understanding of how actin filaments are assembled in cells, our understanding of how such filaments are disassembled remains highly incomplete^10,72,73^. Here, we provide an instrumental piece in the understanding of how filaments are disassembled, by characterizing a family of actin disassembly enzymes conserved from invertebrates to humans. In particular, members of the MICAL family of proteins, which includes one family member in invertebrates and three members in vertebrates including humans, are broadly expressed and have recently emerged because of their crucial role in directing numerous actin-based cellular events *in vivo* (reviewed in^18,22–25^). Using the sole Drosophila member of the MICAL family as a model, we have uncovered that Mical is an F-actin dismantling protein that uses a new enzymatic oxygen-based signaling mechanism to both disassemble actin filaments and limit their reassembly^11,13,14^. Now, we have worked out conditions to purify each of the human MICALs and find that each member of the MICAL family of proteins directly associates with and dismantles actin filaments. These MICAL-mediated effects occur via the ability of actin filaments to directly activate the enzymatic activity of MICAL family members – such that each of the MICAL family enzymes then post-translationally oxidizes actin subunits to destabilize filaments and decrease actin polymerization. Our results also demonstrate that these effects occur both *in vitro* with purified proteins and in cellular contexts *in vivo* – and thereby identify the MICALs as a phylogenetically-conserved family of F-actin dismantling enzymes with important *in vivo* implications for actin regulation because of their broad expression in multiple different tissues.

Our results also identify unique biochemical attributes that define the MICAL family of enzymes. In particular, we find that each MICAL family member binds FAD, is enzymatically active, and behaves catalytically in a generally similar manner – including using NADPH as a co-enzyme, which the MICALs prefer over the related (unphosphorylated) pyridine nucleotide co-enzyme NADH. Likewise, we find that actin filaments activate the catalytic activity of each member of the MICAL family of proteins and serve as a substrate for each MICAL. Furthermore, we find that each of the MICALs uses the same mechanism to accomplish its dismantling effects on actin filaments: the stereospecific oxidation of specific methionine residues within the D-loop portion of actin subunits – effects that are reversed by SelR/MsrB family methionine sulfoxide reductases. Thus, each MICAL family member is selective in its effects – such that it has a specific protein substrate that activates it, particular amino acid residues that it specifically modifies within that substrate, and a stereospecificity in how it modifies its amino acid substrate residues. These attributes, therefore, distinguish MICAL family enzymes from other physiologically-relevant redox enzymes characterized to date such as NADPH oxidases and nitric oxide synthases, which have different mechanisms of activation and action, as well as broader specificity both with regards to protein substrates and the amino acids that are modified within those substrates.

Our results also elucidate that a characteristic feature of each of the MICALs is that actin is their oxygen-acceptor substrate – and that specific binding of the polymeric form of this substrate (i.e., actin filaments) triggers MICALs consumption of NADPH to oxidize individual actin subunits. Thus, from both a structural perspective^12,63–65^, and in the way that binding of the oxygen-acceptor substrate accelerates consumption of the NADPH co-enzyme, MICALs are most similar to flavoprotein monooxygenase enzymes. However, to the best of our knowledge MICAL enzymes are the first known class of flavoprotein monooxygenases for which a protein substrate has been identified. Our results therefore raise the possibility that other (or perhaps all) flavoprotein monooxygenases have protein substrates that are awaiting identification. Moreover, it is unknown if the MICALs have other substrates, but our results herein reveal that a substrate for the MICALs should be defined based solely on its ability to both 1) activate MICAL and 2) be modified by MICALs enzymatic activity (i.e., as we have found for actin filaments). Such a definition will thus serve to differentiate a *bona fide* substrate from a protein that is simply spuriously modified by the ability of MICALs and other flavoprotein monooxygenases to produce non-selective diffusible oxidants (i.e., hydrogen peroxide) in the course of their reaction *in vitro*.

Our results also reveal that like other monooxygenases, the production of hydrogen peroxide (i.e., the loss of reducing equivalents in the absence of a substrate) is actively suppressed by MICAL family enzymes. However, there is some production of hydrogen peroxide, particularly in the case of MICAL-1, with its naturally-occurring single amino acid substitution that we have defined in the critical DG conserved motif of the MICAL enzymatic region. Thus, while our results herein, coupled with previous observations^11,13,14,37^ demonstrate that hydrogen peroxide production and MICAL-mediated F-actin disassembly are not linked together (see also Supplementary Figure 10), it is interesting to consider that MICAL may use hydrogen peroxide in physiological contexts to exert effects on other proteins. In any case, the effects that the MICALs may have using hydrogen peroxide are likely to be on cysteine residues, since hydrogen peroxide is a poor oxidizer of methionine residues^25,74^. Indeed, the oxidation of cysteine residues via hydrogen peroxide has been reported as a means through which MICAL-1 modifies the CRMP protein^27^. It should also be considered that in contrast to our work herein using truncated/active forms of MICAL, full-length MICALs (including MICAL-1) are kept in an inactive state (i.e., without NADPH activity/hydrogen peroxide production)^11,19,28,37,62^. Thus, any hydrogen peroxide production by MICALs is likely to be tightly controlled in the cell to modify proteins in the vicinity of F-actin. Future work will aim to further elucidate the mechanistic details of these unusual and important enzymes, including the means by which the MICALs gain access to and oxidize Met44 and Met47 within filaments (Supplementary Figure 10).

In summary, we have identified a family of actin regulatory proteins conserved from invertebrates to humans that use actin filaments as their enzymatic substrate – employing their unusual catalytic mechanism to covalently modify and induce the dismantling of actin filaments. These findings therefore uncover a new class of broadly-expressed negative regulators of actin stability and thereby elucidate new mechanisms underlying actin disassembly. In particular, the assembly of actin filaments is favored within cellular contexts, making it critical to understand how targeted and rapid F-actin disassembly is occurring^10,72,73^. In a similar way, specific cell-cell signaling ligands including repellents such as ephrins, slits, semaphorins, myelin-associated inhibitors, and Wnts trigger the rapid disassembly of F-actin networks in multiple tissues^20,67,75^, but their direct effectors are still poorly understood. Our results shed light on both of these phenomena, since MICALs are heavily alternatively spliced – with different forms being targeted to specific regions of cells and organelles, as well as working both together and independently from specific repellent cues (reviewed in^18,22–25^). Indeed, a model is emerging that the MICALs are maintained in an inactive conformation in the cell and are locally activated upon binding to other proteins such as the Semaphorin repellent receptor Plexin and small GTPases like Rab35 (Supplementary Figure 10^11,12,19,28,37,62^). Moreover, because of the MICALs ability to covalently modify actin, the MICALs differentiate themselves from other actin disassembly proteins characterized to date because they not only rapidly dismantle F-actin, but they also generate post-translationally modified actin that has aberrant assembly properties. Given that the MICALs are widely expressed in multiple different tissues, that they have the ability to synergize with ubiquitous actin regulatory proteins and signaling pathways, and that they (and their substrate residues on actin) have been tied to different pathologies, our results bring a new understanding to how targeted and rapid F-actin disassembly occurs in cells.

## Materials and Methods

### Molecular Biology and Protein Purification

For all protein expression, plasmids were transformed into ArcticExpress bacterial competent cells (Stratagene, La Jolla, CA, USA) using general approaches^76^. For a given plasmid, a single clone was then inoculated into a 150 ml TB culture medium (including 100 μg/ml ampicillin, 2 mM MgSO_4_, and 20 μg/ml gentamycin) and shaken at 37 °C overnight. 25 ml of overnight culture was then transferred to six 2.8 L flasks containing 1 L TB media (with 100 μg/ml ampicillin and 2 ml of Antifoam B emulsion [Sigma, A6707-500ml]). Flasks were then cultured at 30 °C for ~8 hrs with shaking at 215 rpm. After ~8 hrs, the temperature of the incubator was changed to 10 °C and Isopropyl-β-D-thiogalactoside (IPTG) was added to each of the cultures to a final concentration of 0.5 mM, and each of the cultures were then shaken for 24 hrs. The bacteria was then collected by centrifugation at 2623 × g for 30 min and bacterial pellets were fast frozen in liquid nitrogen and stored at −80 °C until use. The frozen bacterial pellets were thawed at RT and 100 ml of Lysis buffer (50 mM Tris-HCl, 500 mM NaCl, 3 mM β-mercaptoethanol, 20 mM imidazole, 1 tablet of Roche Complete^®^ EDTA-free Protease Inhibitors Cocktail) was added to the combined pellets for a given construct and stirred until the pellet was completely dissolved. The dissolved sample was then loaded into a high-pressure homogenizer (EmulsiFlex-C5, Avestin, Inc., Ottawa, Ontario, Canada) pre-cooled for 1 hr using a refrigerated circulating bath set at 4 °C, and the bacteria were broken by increasing the pressure to 40 psi. In all cases, the output pressure was within 5,000–10,000 psi and the temperature of the sample was controlled by either immersion of the machine/tubing in ice or cooling and applying water using a re-circulating water bath. Initially, the cells were swirled during lysis to prevent cells from getting stuck in the machine and the lysis step was repeated two to three times (up to five times). As an alternative, the bacteria were lysed using a Misonix Ultrasonic Liquid Processor or similar and sonicating for 15 min at 50 amplitude, 5 s on and 5 s off. The bacterial sample in lysis buffer was then centrifuged at 22,000 x *g* for 2 hrs. The supernatant was transferred to a new tube and centrifuged for another 30 min at the same speed. The supernatant was then filtered with a 0.45 μm filter.

To construct human (h) MICAL-1^redoxCH^, a portion of the human MICAL-1 cDNA coding for the redox and CH domains was PCR amplified using primers containing a 5′ SalI restriction enzyme site (Forward: 5′-AGCTGTCGACGGTACCTCTAGCATGGCTTCACCTACCTCCAC-3′) and a 3′ XhoI restriction enzyme site (Reverse: 5′-AGCTCTCGAGTCTAGACTTGAAGGCACTGT-3′) and after digestion of the appropriately sized PCR product with SalI and XhoI, the fragment was inserted into the SalI and XhoI sites of the previously generated pET43.1bNG vector^77^. Positive clones were confirmed by digestion with SalI/XhoI and DNA sequencing. Following bacterial inoculation and preparation of the protein sample from bacteria as described above, the hMICAL-1^redoxCH^ protein was purified (see also Supplementary Figure 1). In particular, a 5 ml HisTrapFF1-GE affinity column was equilibrated with Buffer Ni-A (10 mM Tris-HCl, pH8.0, 500 mM NaCl, 5% glycerol, 3 mM β-mercaptoethanol, 20 mM imidazole) and then the sample containing the hMICAL-1^redoxCH^ protein was loaded onto the column. After washing the column with >20 column volumes (CV) of Buffer Ni-A, the sample was eluted from the column using elution buffer Ni-B (10 mM Tris-HCl, pH8.0, 500 mM NaCl, 5% glycerol, 3 mM β-mercaptoethanol, 250 mM imidazole) and collected in 1 ml aliquots. The presence of the MICAL-1^redoxCH^ protein was then examined by SDS-PAGE and tubes enriched with the hMICAL-1^redoxCH^ protein were retained and combined. The pooled protein sample was then prepared for thrombin digestion to remove the Nus solubility tag by first desalting and exchanging the buffer using a HiPrep 26/10 column (GE Healthcare Bio-Sciences Corporation, Piscataway, NJ, USA). After desalting and eluting with Desalting buffer (10 mM Tris-HCl, pH8.0, 50 mM NaCl, 5% glycerol, 1 mM DTT), the eluate was incubated with 100 μl of 10 μg/μl of the thrombin protease overnight in the cold room. After thrombin digestion, a 1 ml HisTrapFF1-GE affinity column was used to remove the Nus tag using Buffer Ni-A and elution Buffer Ni-B as described above. After another desalting step in which the sample was desalted and eluted with Buffer S-A (20 mM NaPO_4_, pH7.5, 10 mM NaCl, 5% glycerol, 1 mM DTT), the samples containing the MICAL-1^redoxCH^ protein were loaded into a MonoS column (Uno S6 column [Bio-Rad] or Mono S 5/50 GL Column [GE Healthcare]), washed with Buffer S-A (20 mM NaPO_4_, pH7.5, 10 mM NaCl, 5% glycerol, 1 mM DTT) and eluted in 1 ml aliquots with Buffer S-B (20 mM NaPO_4_, pH7.5, 1 M NaCl, 5% glycerol, 1 mM DTT). 10 μl from each aliquot/collection tube was then electrophoresed on an SDS-PAGE gel and those corresponding tubes containing hMICAL-1^redoxCH^ protein were combined together. Millipore Amicon Ultra centrifugal filter (Ultracel-50 kDa cutoff) was then used to reduce the volume to around 500 μl and to change the buffer to storage buffer (10 mM Tris pH8.0, 100 mM NaCl, 5% glycerol, 1 mM DTT). The protein sample was then aliquoted and fast frozen with liquid nitrogen and stored in a −80 °C freezer.

To construct human MICAL-2^redoxCH^, a portion of the human MICAL-2 cDNA encoding for the redox and CH domains was PCR amplified using primers containing a 5′ SalI restriction enzyme site (Forward: 5′-AGCTGTCGACGGTACCTCTAGCATGGGGGAAAACGAGGATGAGA-3′) and a 3′ AvrII restriction enzyme site (Reverse: 5′-AGCTCCTAGGTTAGTGGTGGTGGTGGTGGTGCTCGAGTCTAGACCGGAAGAGCTCGTAGAACT-3′) and after digestion of the appropriately sized PCR product with SalI and AvrII, the fragment was inserted into the SalI and AvrII sites of the previously generated pET43.1bNG vector as described above. Positive clones were confirmed by digestion with SalI/AvrII and DNA sequencing. Following bacterial inoculation and preparation of the protein sample from bacteria as described above, hMICAL-2^redoxCH^ protein was purified (see also Supplementary Figure 2). In particular, transformation, induction, pelleting, lysis, Ni^2+^-NTA affinity chromatography, desalting, and thrombin digestion were carried out as described for purification of hMICAL-1^redoxCH^. The protein sample was then loaded a second time onto a 5 ml HisTrapFF1-GE affinity column and washed with Buffer Ni-A and eluted with Buffer Ni-B. The protein sample was then desalted as described for hMICAL-1^redoxCH^ and eluted with Buffer S-A, loaded onto a MonoS column, washed with Buffer S-A, and eluted with Buffer S-B. Collected samples were then processed as described for hMICAL-1^redoxCH^ and stored in a −80 °C freezer.

To construct human MICAL-3^redoxCH^, a portion of the human MICAL-3 cDNA encoding for the redox and CH domains was PCR amplified using primers containing a 5′ SalI restriction enzyme site (Forward: 5′-AGCTGTCGACGGTACCTCTAGCATGGAGGAGAGGAAGCATGAG-3′) and a 3′ XhoI restriction enzyme site (Reverse: 5′-AGCTCTCGAGTCTAGACTTAAACATCTCGTAGAACTG A-3′) and after digestion of the appropriately sized PCR product with SalI and XhoI, the fragment was inserted into the SalI and XhoI sites of the previously generated pET43.1bNG vector as described above. Positive clones were confirmed by digestion with SalI/XhoI and DNA sequencing. Following bacterial inoculation and preparation of the protein sample from bacteria as described above, hMICAL-3^redoxCH^ protein was purified (see also Supplementary Figure 3). In particular, to purify hMICAL-3^redoxCH^ protein, Ni^2+^-NTA chromatography was performed as described above using a 5 ml HisTrapFF1-GE affinity column with Buffer Ni-A and Buffer Ni-B. The samples containing hMICAL-3^redoxCH^ protein was then desalted as described above using Desalting Buffer, loaded onto a MonoQ column (Mono Q 5/50GL, GE Healthcare), washed with Buffer Q-A (10 mM Tris-HCl, pH8.0, 10 mM NaCl, 5% glycerol, 1 mM DTT), and eluted with Buffer Q-B (10 mM Tris-HCl, pH8.0, 1 M NaCl, 5% glycerol, 1 mM DTT). As described above for hMICAL-2^redoxCH^ protein, the sample was then subjected to thrombin digestion, loading onto a 5 ml HisTrapFF1-GE affinity column, and desalting and purification using a MonoS column. Collected samples were then processed as described for hMICAL-1^redoxCH^ and hMICAL-2^redoxCH^ and stored in a −80 °C freezer.

To construct the human MICAL-1^redoxCH^ D for A substitution (hMICAL-1^DG^), a portion of human MICAL-1^redoxCH^ was PCR amplified using primers containing a 5′ SalI restriction enzyme site (Forward: 5′-AGCTGTCGACGGTACCTCTAGCATGGCTTCACCTACCTCCAC-3′) and a 3′ AatII restriction enzyme site (Reverse: 5′-TGAATTTGACGTCCTTATCTCGGCTGATGGAGGTAAATTCGTCCCTGAAGGCTTC-3′) and inserted into the TopoTA vector (Invitrogen) using the manufacturers recommended protocol. The resulting vector/insert was then cut with the restriction enzymes AatII and SpeI. Then, another portion of human MICAL-1^redoxCH^ was PCR amplified using a 5′ primer containing an Aat II restriction enzyme site and a mutation of the AG residue of the DG motif to a DG residue (Forward Primer: 5′-TGAATTTGACGTCCTTATCTCGGCTGATGGAGGTAAATTCGTCCCTGAAGGCTTC-3′) and a 3′ primer containing both XhoI and Spe I restriction enzyme sites (Reverse Primer: 5′-AGCTACTAGTCTCGAGTCTAGACTTGAAGGCACTGT-3′). The resulting PCR product was then digested with AatII and SpeI and inserted into the corresponding digested sites in the previously generated TOPO vector/insert. This newly generated MICAL-1^redoxCH^ DG cDNA was then cut out of the TopoTA vector with SalI and XhoI and inserted into the SalI and XhoI sites of the previously generated pET43.1bNG vector as described above. Positive clones were confirmed by digestion with SalI/XhoI and DNA sequencing. Following bacterial inoculation and preparation of the protein sample from bacteria as described above, MICAL-1^redoxCH^ DG protein was purified (see also Supplementary Figure 8). In particular, to purify MICAL-1^redoxCH^ DG protein, identical approaches were followed to that described for hMICAL-1^redoxCH^ protein, except an anion ion exchange chromatography was used for the final purification step including a MonoQ column (Mono Q 5/50GL, GE Healthcare), Q-A buffer, and Q-B buffer to elute the sample. Collected samples were then processed as described for hMICAL-1^redoxCH^, hMICAL-2^redoxCH^, and hMICAL-3^redoxCH^ and stored in a −80 °C freezer.

### UV-visible Spectroscopy and Related Analyses

Either a NanoDrop spectrophotometer (Thermo Scientific, Wilmington, DE, USA) or fluorescence spectrophotometer (Spectra Max M2; Molecular Devices, Sunnyvale, CA, USA) was used for all assays and scanning wavelengths between 250 nm and 700 nm were used. To determine whether the flavin was bound covalently or non-covalently and whether FMN or FAD was bound, analyses of each of the MICAL proteins was done using standard approaches and heat-induced denaturation^76,78^. In brief, 2 µl of purified dMical^redoxCH^, hMICAL-1, 2 and 3^redoxCH^ and hMICAL-1 DG proteins were used for obtaining an absorbance spectrum with a NanoDrop spectrophotometer. 100 µl of purified MICAL proteins was used to monitor absorption spectra using a fluorescence spectrophotometer. 200 µl of 10 mM HEPES/NaOH, 50 mM NaCl buffer, pH 7.5 was added into each tube to dilute samples. The 5 tubes with samples were incubated for 10 min at 100 °C. After being cooled on ice, microfuge tubes are centrifuged at 4 °C for 10 min at 13,000 rpm (14,500 g). The supernatant was recovered and the absorbance spectrum was then recorded. The original data were then exported into Excel software (Microsoft Corp., Redmond, WA, USA) and presented using GraphPad software (La Jolla, CA, USA), to compare the difference between purified and denatured proteins.

Calculation of the percentage of FAD bound to purified MICAL proteins (i.e., the stoichiometry of FAD to MICAL proteins) was done using standard approaches^76,79,80^ such that the concentration of each of the purified MICAL^redoxCH^ proteins was determined as described above by adding 2 μl to the platform of a NanoDrop spectrophotometer and measuring the absorption at 280 nm. The concentration of FAD in the purified sample was then determined using standard approaches^76,78^, by denaturing the MICAL^redoxCH^ proteins with 0.2% SDS, pelleting the denatured MICAL^redoxCH^ proteins, measuring the absorbance of the free FAD in the sample, and then using the Beer-Lambert law (Absorption at 459 nm = ε [extinction coefficient, also known as molar absorptivity] × C [concentration in M] × l [path length in cm of the cuvette in which the sample is contained]) to calculate the concentration of FAD. The concentration of FAD in the purified sample was then divided by the concentration of the MICAL^redoxCH^ proteins in the purified sample to determine the percentage (stoichiometry) of FAD bound to the purified MICAL proteins.

### NADPH and NADH Consumption

NADPH and NADH (the reduced form of the coenzymes) absorb light at 340 nm, while the oxidized forms (NADP^+^ and NAD+) do not. This difference between the oxidized and reduced forms of the coenzymes makes it straightforward to measure the conversion of one to another in enzyme assays. Thus, the enzymatic activity of Mical was monitored by the rate of NADPH or NADH oxidation, which is measured by the rate of decreasing light absorbance at 340 nm (extinction coefficient 340 = 6.2 mM^−1^*cm^−1^). 600 nM of the different MICALs to 100 µM NADPH in General Actin Buffer was used. In the absence of F-actin (Fig. 1g,h), NADPH and NADH consumption was monitored and reported as described^76^. In the presence of F-actin (Figs 3a, 5d), NADPH consumption was monitored and reported as described^13,16^. In particular, the basal NADPH consumption was measured in the presence of different concentrations of F-actin for the first 3 min before adding the MICALs. The MICAL enzymatic activity was determined by subtracting the NADPH consumption after addition of the MICAL^redoxCH^ from basal NADPH consumption. The rate of NADPH consumption was determined by 10 sec intervals at the maximum rate.

### MICALs and F-actin Co-sedimentation Assays

Standard approaches in multiple independent experiments (n > 3) were used for high-speed sedimentation/co-sedimentation assays (^11,13,16,81^; Cytoskeleton, Inc). In brief, purified non-muscle actin (85% β-actin, 15% γ-actin; Cytoskeleton, Inc.) was resuspended to 1 mg/ml in a general actin resuspension buffer (5 mM Tris-HCl pH8.0, 0.2 mM CaCl_2_). The resuspended actin was then added to a standard actin polymerization buffer (50 mM KCl, 2 mM MgCl_2_, and 1 mM ATP) and allowed to polymerize for 1 hour at room temperature. This generated an F-actin stock at 23 μM actin. dMical^redoxCH^ protein, hMICAL^redoxCH^-1, hMICAL^redoxCH^-2, hMICAL^redoxCH^-3, hMICAL^redoxCH^-1 DG protein, or a negative control (bovine serum albumin (BSA), Cytoskeleton, Inc) were subjected to initial (clarification) high-speed centrifugation at 150,000 x g for 1 hour at 4 °C. Test proteins (at a final concentration of 2 μM) were then added to separate tubes and incubated with either F-actin (at a final concentration of 18.4 μM) or with F-actin buffer only for 30 min at room temperature. An F-actin only tube was also incubated for 30 minutes at room temperature. All test tubes were then subjected to high-speed centrifugation at 150,000 × g for 1.5 hours at 24 °C. Supernatants were carefully removed and added to sample buffer for loading on an SDS-PAGE gel. The pellet was resuspended in Milli-Q H_2_O with pipetting, incubation on ice for 10 min, and then more pipetting before being added to sample buffer for loading on an SDS-PAGE gel. The gel was then stained with Coomassie blue using standard approaches. The intensity of each of the stained bands in the pellet and soluble fraction was then analyzed and quantified by densitometry using Image J (NIH) and the percentage of different purified proteins with F-actin in the pelleted fraction was presented. Similar approaches and as described previously for Drosophila Mical^redoxCH^ were also used for mutant actins^13^.

### Microtubule Co-sedimentation and Polymerization Assays

Tubulin was obtained from Cytoskeleton, Inc and standard approaches were used for the microtubule co-sedimentation assays (^82,83^; Cytoskeleton, Inc) and as we have previously employed for Drosophila Mical^redoxCH^ ^11^. In brief, microtubules were generated by polymerizing tubulin (from a 5 mg/ml tubulin stock containing 1 mM GTP; Cytoskeleton, Inc) at 35 °C for 20 min in a polymerization buffer (80 mM PIPES pH 6.9, 0.5 mM EGTA, 2 mM MgCl_2_, 7.5% glycerol). Microtubules was then diluted 10 fold in a warm buffer (35 °C) containing 80 mM PIPES pH 6.9, 0.5 mM EGTA, 2 mM MgCl_2,_ and 20 μM taxol. Test proteins including hMICAL^RredoxCH^-1, hMICAL^redoxCH^-2, hMICAL^redoxCH^-3, hMICAL^redoxCH^-1 DG protein (1 μM final concentration), a negative control (bovine serum albumin (BSA) [2.2 μM final concentration, Cytoskeleton, Inc.]), and a positive control (microtubule associated proteins MAPs [0.64 μM final concentration; Cytoskeleton, Inc.]) were added to separate tubes and incubated with either microtubules or buffer for 30 min at room temperature. A microtubule only tube was also incubated for 30 min at room temperature. Each reaction was then subjected to high-speed centrifugation at 100,000 × g for 40 min at 24 °C as described by Cytoskeleton, Inc. Supernatants were then carefully removed and added to sample buffer for loading on an SDS-PAGE gel. The pellet was resuspended in Milli-Q H_2_O with pipetting before being added to sample buffer for loading on an SDS-PAGE gel. The distribution of microtubule and test proteins were visualized with Coomassie blue staining and the intensity of each of the stained bands in the pellet and soluble fraction was quantified by densitometry using Image J (NIH) and the percentage of different purified proteins with microtubules in the pelleted fraction was presented as we have previously described^11^. Likewise, the effects of the hMICAL^redoxCH^ proteins on microtubule polymerization were measured using fluorescence-based standard approaches (^84^; Cytoskeleton, Inc) and as we have previously employed for Drosophila Mical^redoxCH^ ^11^. In brief, tubulin (bovine tubulin; Cytoskeleton, Inc.) polymerization was performed in a microtubule polymerization buffer (80 mM PIPES pH 6.9, 2 mM MgCl_2_, 0.5 mM EGTA, 15% glycerol, 1 mM GTP and 5 μM fluorescent reporter (DAPI)) containing mixed tubulin (2 mg/ml final concentration), dMical^redoxCH^ protein, hMICAL^redoxCH^-1, hMICAL^redoxCH^-2, hMICAL^redoxCH^-3, hMICAL^redoxCH^-1 DG protein (0.6 μM final concentration) and/or NADPH (100 μM). The polymerization was initiated by raising the temperature from 4 °C to 37 °C. Fluorescence intensity was monitored for 1 hour at 450 nm with excitation at 360 nm by a fluorescence spectrophotometer (Spectra max M2; Molecular Devices) with temperature control.

### Actin Polymerization and Depolymerization Assays

Pyrene-actin polymerization assays were performed using standard approaches^85^ (Cytoskeleton, Inc.) and as we have previously employed for Drosophila Mical^redoxCH^ ^11,13,16,76,86^. In brief, purified rabbit skeletal muscle actin (pyrene-labeled; Cytoskeleton, Inc) was used to monitor actin polymerization since the fluorescence intensity of the pyrene-labeled polymer is substantially higher than the pyrene-labeled monomer. G-actin (monomeric actin) was resuspended to 9.2 μM in a G-actin buffer (5 mM Tris-HCl pH 8.0, 0.2 mM CaCl_2_, 0.2 mM ATP and 1 mM DTT) and incubated on ice for 1 hour. Before all the experiments, G-actin solution was centrifuged for 1 hour at 100,000 × g at 4 °C to remove residual actin nucleating centers. Multiple independent experiments (n > 3 by two independent researchers) were performed for each condition such that MICAL proteins, NADPH (MP Biomedicals), and/or NADH (MP Biomedicals) were then added to the actin in a 96 well plate and polymerization was initiated (Time = 0) at 25 °C by the addition of 5 mM Tris-HCl pH 7.5, 50 mM KCl, 2 mM MgCl_2_, 1 mM EGTA, 0.5 mM DTT, and 0.2 mM ATP (to the 96 well plate with 2–5 seconds gentle shaking using the shaking feature of the fluorescence spectrophotometer). Actin was used at a final concentration of 1.1 μM. Fluorescence intensity was immediately monitored at 407 nm with excitation at 365 nm by a fluorescence spectrophotometer (Spectra max M2; Molecular Devices).

To examine the ability of MICALs to induce depolymerization in conditions that favored polymerization (see also^11^), multiple independent experiments (n > 3 by two independent researchers) were performed similar to described previously for both pyrene-actin and non-pyrene wild-type and Actin M4447L sedimentation assays^11,13,16,86^. MICAL proteins and/or NADPH were then added to the polymerized actin as described for the polymerization assays (Time = 0) and depolymerization was immediately monitored by fluorescence intensity or via sedimentation as described above. In some cases, as described in the results/figures, MICAL proteins and NADPH were added together before adding them to the F-actin (pre-actin incubation) and in some cases, MICAL proteins were added to F-actin in which NADPH had already been added (post-actin incubation). In some cases, as described in the results/figures, additional NADPH was added to the F-actin/MICAL mix at a later time. Note also that these experiments were done using standard approaches and that the F-actin was not stabilized (i.e., by adding a stabilizing protein).

### Analysis of MICAL-oxidized actin

Actin was polymerized to generate F-actin and 600 nM of each of the human MICALs and 200–300 µM of NADPH were added to 1.15 μM F-actin at room temperature for 2 hours. The MICAL/NADPH/F-actin reaction was then stopped by adding loading buffer containing β-mercaptoethanol and boiling samples for 5 min. All samples were then loaded into a 12% SDS-PAGE gel, transferred to PVDF membrane, blocked with 5% non-fat milk/TBST buffer for 1 hour and then incubated for 1 hour with a 1:500 dilution of the actin MetO44 antibody^16^.

### SelR-treatment of MICAL-oxidized Actin

Purified pyrene-labeled rabbit skeletal muscle actin (Cytoskeleton, Inc.) was resuspended in G-actin buffer to 2.3 μM. The resuspended actin was then polymerized with 2X polymerization buffer (1.15 μM actin = final concentration) in the presence of human MICAL-1^redoxCH^, human MICAL-2^redoxCH^, human MICAL-3^redoxCH^, or human MICAL-1^redoxCH^ DG and 100 μM NADPH for 1 hour. The NADPH was then removed from the human MICAL-treated actin as described previously^13,14^ (using a centrifugal filter [Amicon Ultra, Ultracel-10K, Millipore]). The human MICAL-treated actin was then either treated with SelR or the buffer the SelR was stored in (containing the 10 mM MgCl_2_ and 20 mM DTT) for 1 hour at 37 °C.

### Hydrogen Peroxide Production

In one method, we used the substrate luminol and the generation of a chemiluminescence signal that we visualized on either X-ray film or a phosphoimager. In particular, luminol, when it becomes oxidized by peroxide (peroxide can be formed through a reaction of H_2_O_2_ with horseradish peroxidase [HRP]), results in creation of an excited state product, which then decays to a lower energy state by releasing photons of light^87^. Therefore, to perform this reaction and to test for the ability of the different MICALs to form H_2_O_2_, we added a substrate (luminal [i.e., ECL substrate]), a catalyst (HRP), and the MICALs in the presence of NADPH (which will provide H_2_O_2_ if they generate it). To do this, we incubated 0.6 μM of each MICAL in the presence of 200 μM NADPH, HRP (the catalyst), and luminol, and then visualized the product of the reaction using either X-ray film or a phosphoimager.

As another (different) means to detect H_2_O_2_, we used the ROS-Glo H_2_O_2_ Assay Kit developed by Promega (Catalog No: G8820^88^), and followed the manufacturers recommended instructions. In particular, in the ROS-Glo H_2_O_2_ Assay Kit, a derivatized luciferin substrate is incubated with a potential H_2_O_2_ generating sample, and then the derivatized luciferin substrate reacts directly with H_2_O_2_ to generate a luciferin precursor. Addition of the ROS-Glo detection solution converts the precursor to luciferin and triggers the luciferase to produce a light signal that is proportional to the level of H_2_O_2_ present in the sample. Thus, the ROS-Glo H_2_O_2_ substrate reacts directly with H_2_O_2_, eliminating the need for HRP as a coupling enzyme (and thus eliminating any false detection of H_2_O_2_ associated with any unknown activation or inhibition of HRP by the MICALs). In particular, 60 pmol of each of the Drosophila and human MICAL^redoxCH^ proteins was diluted by General Actin Buffer to a final volume of 79 µl and transferred into a 96-well plate (Corning). 25 µM of H_2_O_2_ was used as the standard. Following the manufacturer′s recommended protocol, the H_2_O_2_ substrate dilution buffer (SDB) was thawed and placed on ice. The SDB was then mixed with the manufacturer-provided H_2_O_2_ substrate (the derivatized luciferin substrate) just prior to use (generating the SDB-S). 20 µl of SDB-S was added into each well containing the MICALs or the H_2_O_2_ standard to generate a final volume of 100 µl. A control SDB-S only well was also used. At time 0 min (reaction time: 10 min), 5 min (reaction time: 5 min), 8 min (reaction time: 2 min), 1 µl of 10 mM NADPH (final concentration: 100 µM) was added into each well. 100 µl of ROS-Glo detection solution (+1 µl D-Cys and 1 µl enhancer solution/100 µl Detection solution) was added to each well. After the 96-well plate was incubated for 5 min at room temperature, the relative luminescence unit was recorded by using a plate-reading luminometer (TriStar² LB 942 Multidetection Microplate Reader, Berthold Technologies, Germany). The H_2_O_2_ concentration was calculated by the luminescence value of the samples, zero control and the standard sample (25 µM H_2_O_2_).

It should also be noted that the Amplex Red Hydrogen Peroxide/Peroxidase Assay Kit (Invitrogen) has been used in the past to determine the amount of H_2_O_2_ generated by MICAL-1^19,64^. We and others have determined that this Amplex Red reagent is artifactually fast and an inaccurate measure of MICAL-mediated H_2_O_2_ production^89,90^. It should be noted, however, that we did see similar relative amounts of “H_2_O_2_” generated with this reagent (i.e., MICAL-1 generated the most reaction product and MICAL-2 generated the least) when we used this reagent to compare each of the MICALs (data not shown).

### Oxygen Consumption

The electrode was prepared by adding 50% saturated KCl solution as electrolyte in the electrode well. The well was then covered with a paper spacer to provide a continuous electrolyte layer on the electrode and with an oxygen-permeable membrane on top of the paper spacer to separate the electrolyte from the sample. Then a glass reaction vessel was placed on top of the electrode chamber and closed with a gas-tight plunger to provide an almost sealed space for the sample with a small opening on top to inject reagents. Lastly, the electrode was calibrated using dissolved oxygen in water (which provided a basal line as a sample) and then establishing a zero content of oxygen by adding an oxygen reducing agent to the water. The basal oxygen consumption was then determined by adding 200 µM of NADPH and 600 nM of each MICAL into F-actin buffer (no F-actin) (1:1 G-Buffer+2X Polymerization Buffer) in the chamber and measuring the change in the amount of oxygen dissolved in the buffer via the oxygen electrode.

### Drosophila Transgenic Fly Lines

To generate hMICAL-1^redoxCH^ pUAST, the following primers were used: EcoRI-For: 5′-AGCT GAATTC ATG GCT TCA CCT ACC TCC AC-3′ and NotI-Rev: 5′- AGCT GCGGCCGC TTA GTG GTG GTG GTG GTG GTG CTC GAG TCT AGA CTT GAA GGC ACT GTG GAA GTG -3′. Both PCR product and the pUAST vector were digested with EcoRI and NotI and the purified DNA fragments were ligated and sequenced on both strands. To generate hMICAL-2^redoxCH^ pUAST, the following primers were used: NotI- For: 5′-AGCT GCGGCCGC ATG GGG GAA AAC GAG GAT GAG A -3′ and NheI- Rev: 5′-AGCT GCTAGC TTA GTG GTG GTG GTG GTG GTG CTC GAG TCT AGA CCG GAA GAG CTC GTA GAA CT-3′. Both PCR product and the pUAST vector were digested with NotI and NheI (pUAST digested with XbaI) and the purified DNA fragments were ligated. To generate hMICAL-3^redoxCH^ pUAST, the following primers were used: NotI-pUAST-For: 5′-AGCT GCGGCCGC ATG GAG GAG AGG AAG CAT GA-3′ and KpnI- Rev: 5′-AGCT GGTACC TTA GTG GTG GTG GTG GTG GTG CTC GAG TCT AGA CTT AAA CAT CTC GTA GAA CTG A-3′. Both PCR product and the pUAST vector were digested with NotI and KpnI and the purified DNA fragments were ligated.

### *In Vivo* F-actin and Cellular Assays

Multiple different transgenic fly lines for each of the MICALs were generated (n > 5) and transgenic fly lines of the same genotypes showed similar defects when expressed with the bristle-specific B11-GAL4 driver. Analysis of the effects on F-actin and cellular remodeling was done *in vivo* using the Drosophila bristle process as previously described^11,14,16,17^. To visualize F-actin, Drosophila pupae were placed on double-sided tape and the dorsal surface of the pupal case was removed, allowing the pupae to be lifted from their case and immediately placed in depression-well slides and imaged. One copy of *UAS:*^*GFP*^*actin* was used to visualize F-actin and imaging was done using a Zeiss LSM510 confocal microscope. Examination of bristle cell remodeling was performed on young, recently emerged adult offspring. The images and drawings of the adult bristles were done with the aid of a Zeiss Discovery M^2^ Bio stereomicroscope, a motorized focus and zoom, and three-dimensional reconstruction software (Extended Focus Software; a kind gift from Bernard Lee).

### Statistics and Reproducibility

For each representative protein purification, image, gel, immunoblot, graph, or *in vivo* experiment, the experiments were repeated at least two separate independent times and there were no limitations in repeatability. At least two independent protein purifications and multiple independent actin biochemical experiments were performed with similar results including reproducing the effects independently from different researchers. No statistical method was used to predetermine the sample size, which was based on what is published in the field. Differences between experimental and control animal conditions were large, with little variability and so the sample size was larger than needed to ensure adequate power to detect an effect. Animal studies were based on pre-established criteria to compare against age-matched animals. Animal experiments were not randomized. Animals of the correct genotype were determined and those collected of that genotype were included as data. For genetic experiments, in which the genotype needed to be determined on the basis of different Drosophila genetic/chromosome markers, blinding was not employed. The figure legends list the sample size for each experiment. To the best of our knowledge the statistical tests are justified as appropriate.

### Data availability

All data generated or analysed during this study are included in this published article (and its Supplementary Information files). All materials, data and associated protocols will be made promptly available to others without preconditions.

## Electronic supplementary material

Supplementary Information
