# Supplementary material for: The MICALs are a Family of F-actin Dismantling Oxidoreductases Conserved from Drosophila to Humans
Source: Sci Rep. 2018 Jan 17;8:937. doi: 10.1038/s41598-017-17943-5 (PMC5772675; doi:10.1038/s41598-017-17943-5)
Supplement: Supplementary file 1 — Supplementary Information [file 41598_2017_17943_MOESM1_ESM.pdf]

Supplementary Information

for

The MICALs are a Family of F-actin Dismantling Oxidoreductases Conserved from Drosophila to Humans

Heng Wu, Hunkar Gizem Yesilyurt, Jimok Yoon, and Jonathan R. Terman

hMICAL-1^{redoxCH} purification

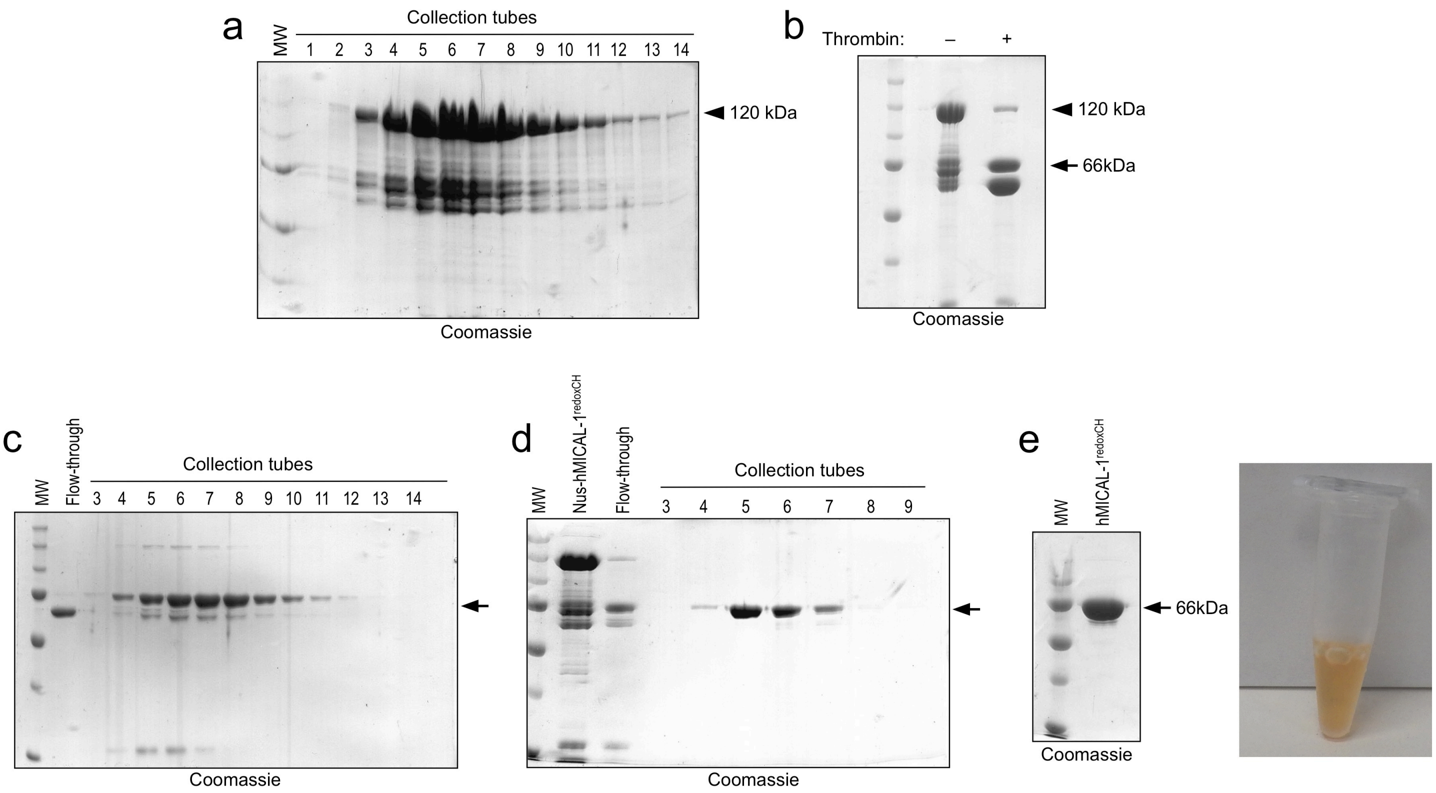

Supplementary Figure 1. Purification and characterization of human MICAL-1^{redoxCH} protein. (a-e) Coomassie stained gels are shown and the arrows point to the recombinant human (h) MICAL-1^{redoxCH} protein in all gels. MW in kDa. **(a)** A cDNA encoding hMICAL-1^{redoxCH} was inserted into a His-tag containing bacterial expression vector, transformed into bacteria, and following the appropriate growth conditions, lysates were loaded on a Nickel (Ni)-NTA affinity column to enrich for the Nus/His-tagged hMICAL-1^{redoxCH} (arrowhead). **(b)** Following elution from the Ni-NTA column, the enriched Nus/His-tagged hMICAL-1^{redoxCH} protein (arrowhead, 120 kDa) was digested (+) with a thrombin protease to cleave-off the Nus tag. The smaller size of the digested human MICAL-1^{redoxCH} (without the Nus tag) can be seen (arrow, 66 kDa). **(c)** The digested (+) sample from (b) was then loaded again on a Ni-NTA column to separate hMICAL-1^{redoxCH} from the Nus tag. Fractions within collection tubes 3-12 were combined. **(d)** Ion-exchange chromatography was then used to remove contaminating proteins since hMICAL-1^{redoxCH} can bind with a MonoS column (arrow). Samples within collection tubes 5-7 were then combined and concentrated **(e)** and analyzed on a gel to determine the purity of the human MICAL-1^{redoxCH} protein (arrow). The purified hMICAL-1^{redoxCH} is also shown in a transparent microcentrifuge tube, where its yellowish color is readily observed. Unprocessed original scans of gels/blots are shown in Supplementary Fig. 11.

hMICAL-2^{redoxCH} purification

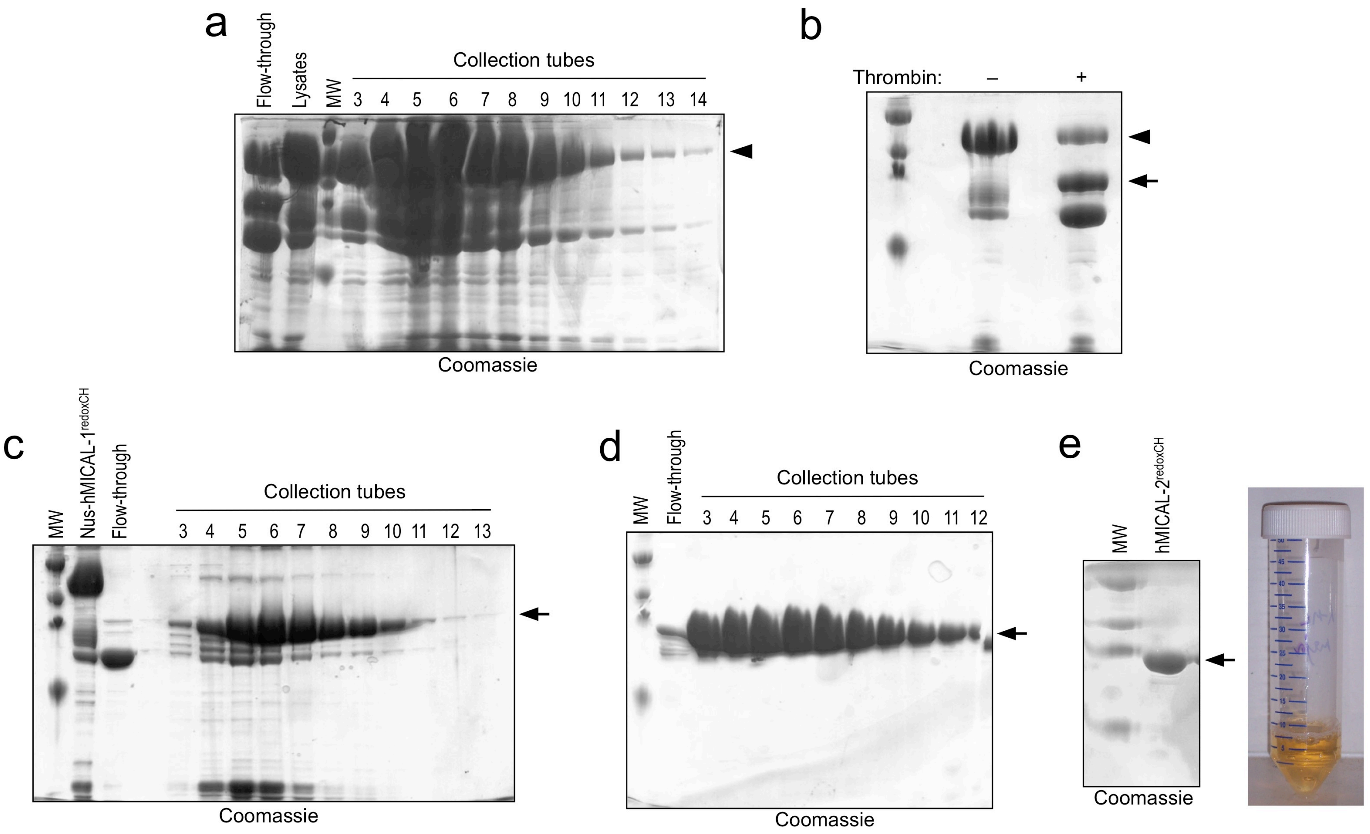

Supplementary Figure 2. Purification and characterization of human MICAL-2^{redoxCH} protein. (a-e) Coomassie stained gels are shown and the arrows point to the recombinant hMICAL-2^{redoxCH} protein in all gels. MW in kDa. **(a)** A cDNA encoding hMICAL-2^{redoxCH} was inserted into a His-tag containing bacterial expression vector, transformed into bacteria, and following the appropriate growth conditions, lysates were loaded on a Ni-NTA affinity column to enrich for the Nus/His-tagged hMICAL-2^{redoxCH} (arrowhead). **(b)** Ni^{2+}-NTA affinity purified Nus-tagged hMICAL-2^{redoxCH} protein from samples in (a) were combined and subjected to thrombin digestion (+) to remove the Nus tag. A Coomassie-stained band corresponding in size to the cleaved hMICAL-2^{redoxCH} protein was observed following thrombin digestion (arrow), while the uncleaved Nus-tagged hMICAL-2^{redoxCH} protein is readily seen in the absence of thrombin (arrowhead). **(c)** Samples containing cleaved hMICAL-2^{redoxCH} protein were subjected to Ni^{2+}-NTA affinity chromatography to remove the Nus-tag and thrombin. hMICAL-2^{redoxCH} protein (arrow) was eluted with 250 mM imidazole and fractions within collection tubes 3-13 were combined for the next step. **(d)** As seen after SDS-PAGE/Coomassie-staining, cation ion exchange chromatography was used to separate hMICAL-2^{redoxCH} protein (arrow) from contaminating proteins, including chaperonins. **(e)** Samples from collection tubes 3-12 (from d) were concentrated and the purity of the hMICAL-2^{redoxCH} is shown after SDS-PAGE and Coomassie staining (arrow). The purified hMICAL-2^{redoxCH} is also shown in a transparent tube, where its yellowish color is readily observed. Unprocessed original scans of gels/blots are shown in Supplementary Fig. 11.

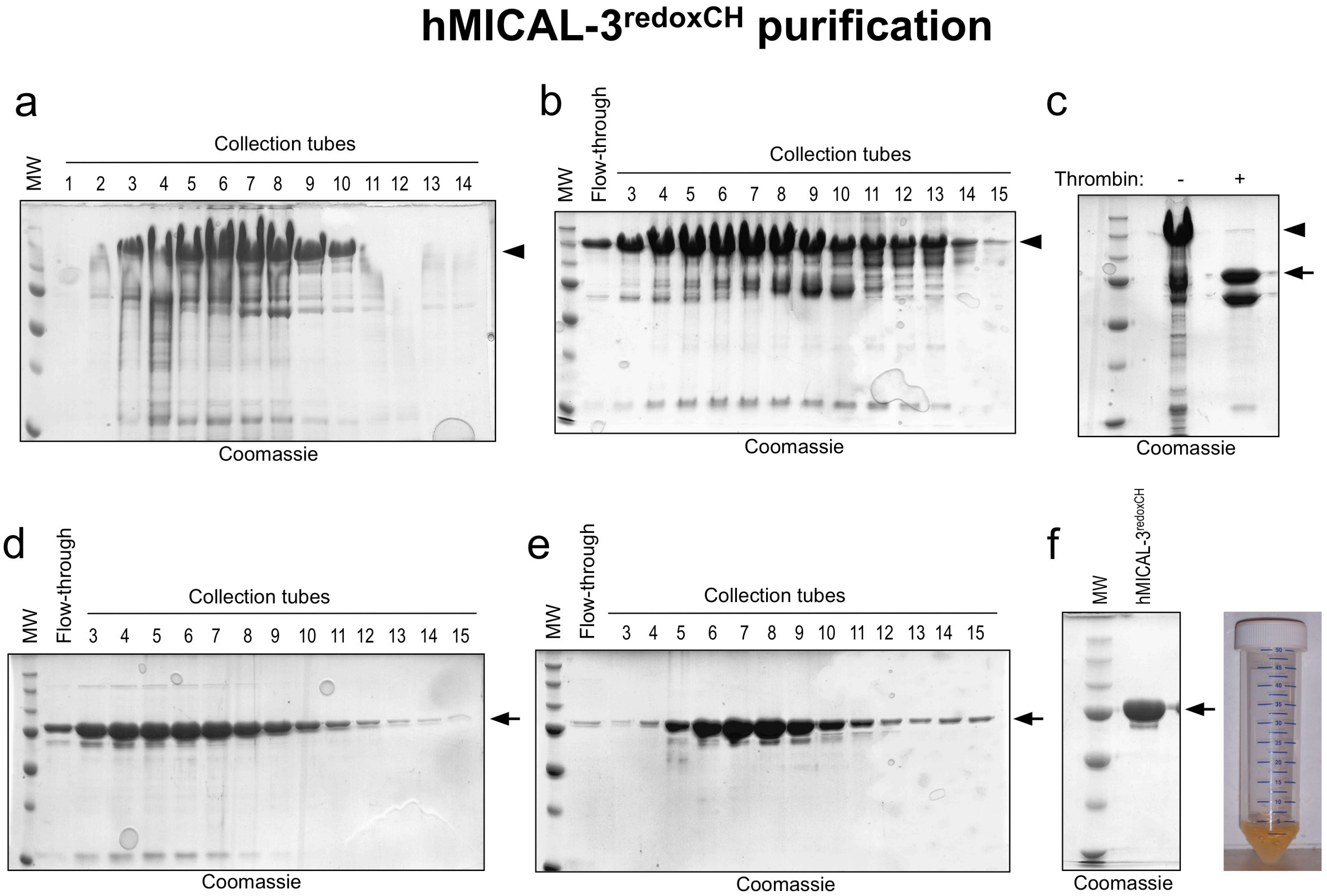

Supplementary Figure 3. Purification and characterization of recombinant human MICAL-3^{redoxCH} protein. (a-f) Coomassie stained gels are shown and the arrows point to the recombinant hMICAL-3^{redoxCH} protein in all gels. MW in kDa. (**a**) A cDNA encoding hMICAL-3^{redoxCH} was inserted into a His-tag containing bacterial expression vector, transformed into bacteria, and following the appropriate growth conditions, lysates were loaded on a Ni-NTA affinity column to enrich for the Nus/His-tagged hMICAL-3^{redoxCH} protein (arrowhead). **(b)** After desalting, samples were loaded on a MonoQ column to enrich for the Nus/His-tagged hMICAL-3^{redoxCH} (arrowhead). **(c)** Samples in collection tubes 3-9 (from b) were desalted and then digested with thrombin. A Coomassie-stained band corresponding in size to the uncleaved Nus-tagged hMICAL-3^{redoxCH} protein is readily seen in the absence of thrombin (arrowhead), while cleaved hMICAL-3^{redoxCH} protein is observed following thrombin digestion (arrow). **(d-e)** Ni^{2+}-NTA affinity chromatography (d) was used again to remove the Nus-tag and thrombin, after which the contents of collection tubes 3-15 were combined and subjected to cation ion exchange chromatography (e) to further separate hMICAL-3^{redoxCH} protein (arrow) from contaminating proteins. **(f)** The contents of sample tubes 4 through 15 (from e) were combined, concentrated, and analyzed on a gel to determine the purity of the hMICAL-3^{redoxCH} protein (arrow). The purified hMICAL-3^{redoxCH} is also shown in a transparent tube, where its yellowish color is readily observed. Unprocessed original scans of gels/blots are shown in Supplementary Fig. 11.

a Identity/Similarity of the Redox Region of MICAL Family Proteins

	dMical^{redox}	hMICAL-1^{redox}	hMICAL-2^{redox}	hMICAL-3^{redox}
**dMical^{redox} (469aa)**				
**hMICAL-1^{redox} (470aa)**	54%(I) 70%(S)			
**hMICAL-2^{redox} (473aa)**	62%(I) 74%(S)	59%(I) 72%(S)		
**hMICAL-3^{redox} (473aa)**	63% (I) 76% (S)	60%(I) 75%(S)	81% (I) 91% (S)	

b Flavin-binding Characteristics of MICAL Family Proteins

	Supe (after Boiling)	Pellet (after Boiling)	*Flavin – Protein Binding*	Peak at 446nm or 450nm?	*FMN or FAD?*
**dMical**	yellow	white	*Non-covalent*	450nm	*FAD*
**hMICAL-1**	yellow	white	*Non-covalent*	450nm	*FAD*
**hMICAL-2**	yellow	white	*Non-covalent*	450nm	*FAD*
**hMICAL-3**	yellow	white	*Non-covalent*	450nm	*FAD*
**hMICAL-1 DG**	yellow	white	*Non-covalent*	450nm	*FAD*

c FAD Stoichiometry (Bound FAD) of MICAL Family Proteins

	Bound FAD (%)
**dMical**	97.03
**hMICAL-1**	99.73
**hMICAL-2**	95.31
**hMICAL-3**	99.76
**hMICAL-1 DG**	79.90

Supplementary Figure 4. The redox region and flavin binding characteristics of MICAL family proteins. Essential experimental procedures for defining a flavin-containing enzyme are 1) the recognition of the presence of a flavin co-factor, 2) the identification of the flavin co-factor, and 3) the determination of the stoichiometry of the bound flavin co-factor [1,2]. Therefore, we set out to characterize each member of the MICAL family in this regard (see also **Figure 1c-f**). **(a)** The length, identity (I), and similarity (S) of the amino acid (aa) sequences coding for the redox portion of Drosophila (d) Mical and each of the human (h) MICALs. **(b)** Heat-induced denaturation of each the MICAL proteins using standard approaches [1] was used to determine the each purified MICAL family protein bound FAD non-covalently. In particular, following heat-induced denaturation of each of the MICAL proteins and centrifugation, the supernatant (Supe) and not the pellet was yellow, indicating that the flavin is bound non-covalently (i.e., the flavin was released when the protein was unfolded by heat treatment/denaturation, showing that it was not covalently linked to the MICALs). The absorbance spectrum of the released flavin exhibited a peak at 450 nm, which is characteristic of FAD. FMN exhibits a peak at 446 nm. These results indicate that each of the MICALs, including hMICAL-1^{DG} (see **Supplementary Figure 8**), binds FAD non-covalently. [MICALs]=20μM. **(c)** Our results also revealed a 1:1 stoichiometry of the bound FAD cofactor to each of the MICAL family members. Characterization of the absorption spectra of MICAL proteins (see **Figure 1c-f**) also allows the amount of the purified MICAL that is bound to FAD to be determined (see Materials and Methods and [3]). Note that the MICAL-1^{DG} protein generated more protein in the Apo form (without FAD) than the other MICALs, indicating that it is functional but less able to bind and incorporate FAD when this amino acid change is introduced into it.

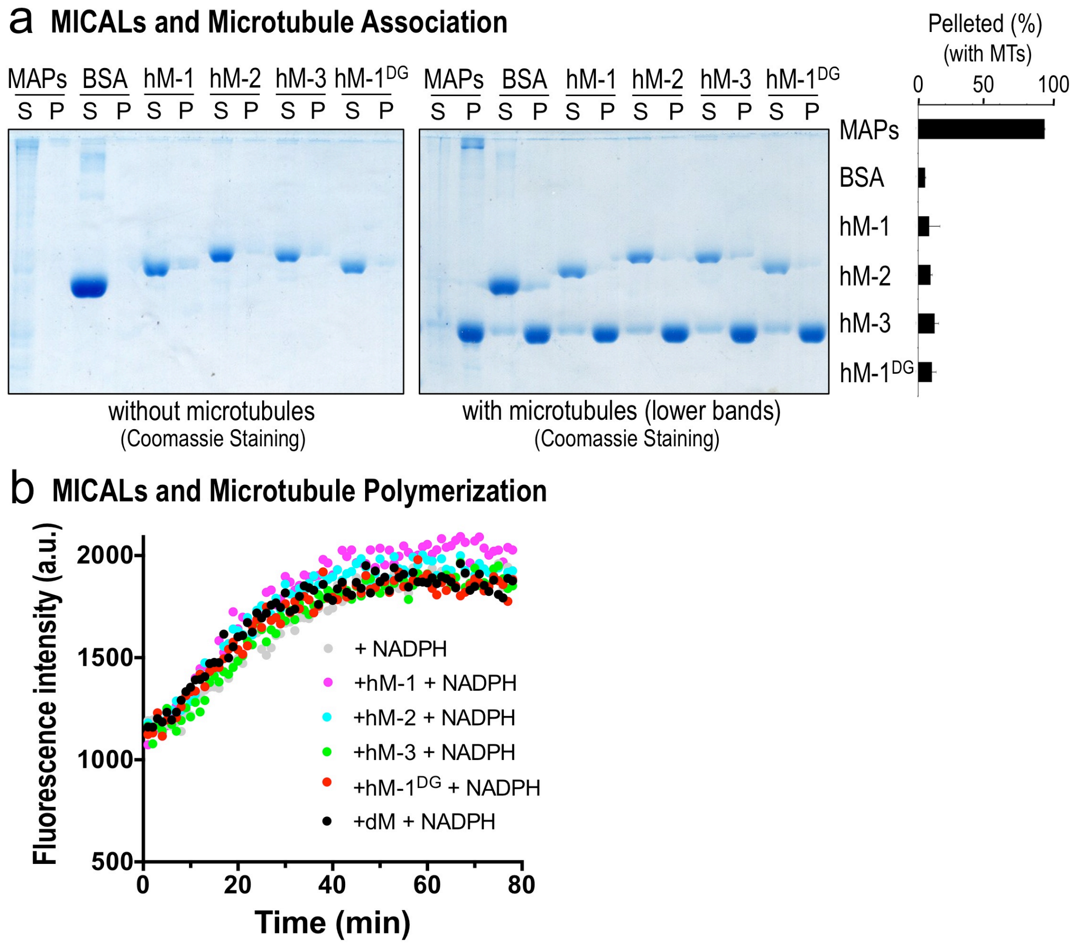

Supplementary Figure 5. Purified human MICAL^{redoxCH} proteins show little to no association with microtubules and do not alter tubulin polymerization dynamics. (a) Images of Coomassie blue stained gels are shown. Co-sedimentation analysis was used to examine the association between MICALs and microtubules. Notice that after high-speed centrifugation, each of the purified human MICAL^{redoxCH} proteins, hMICAL-1 (hM-1), hMICAL-2 (hM-2), hMICAL-3 (hM-3), and human MICAL-1 DG (hM-1^{DG}), like Drosophila Mical^{redoxCH} (dM) [4], is present in the soluble (S) fraction (left gel). Similarly, in the presence of microtubules, the majority of the purified MICAL proteins (and a negative control, BSA) are present in the soluble (S) fraction (right gel). Notice, however, that microtubule associated proteins (MAPs), known microtubule binding proteins that were used as a positive control, change their distribution from the soluble fraction to pellet fraction in the presence of microtubules. These results indicate that purifed human MICAL^{redoxCH} proteins have little to no association with microtubules. The percentage ($\pm$ the standard error of the mean (SEM)) of different purified MICAL proteins in the pelleted fraction following incubation with microtubules (MTs) was quantified by densitometry ($n \geq 2$). [MICALs]=1 µM, [BSA]= 2.2 µM, [MAPs]= 0.64 µM, [tubulin]=0.5mg/ml. **(b)** The effect of MICALs on tubulin polymerization was examined. A fluorescence-based tubulin polymerization assay was employed using standard approaches, where the fluorescence intensity (a.u. (arbitrary units)) of microtubules is substantially higher than tubulin monomers. Drosophila Mical^{redoxCH} (dM), each of the purified human MICAL^{redoxCH} proteins, hMICAL-1 (hM-1), hMICAL-2 (hM-2), hMICAL-3 (hM-3), and human MICAL-1 DG (hM-1^{DG}), and/or NADPH were added in the tubulin solution and polymerization was initiated by increasing the temperature from 4°C to 37°C. There is no appreciable difference between tubulin polymerization alone (+NADPH) and tubulin polymerization in the presence of the human MICALs with NADPH. [MICALs]=600nM, [NADPH]=100µM, [tubulin]=2mg/ml. Unprocessed original scans of gels are shown in Supplementary Fig. 11.

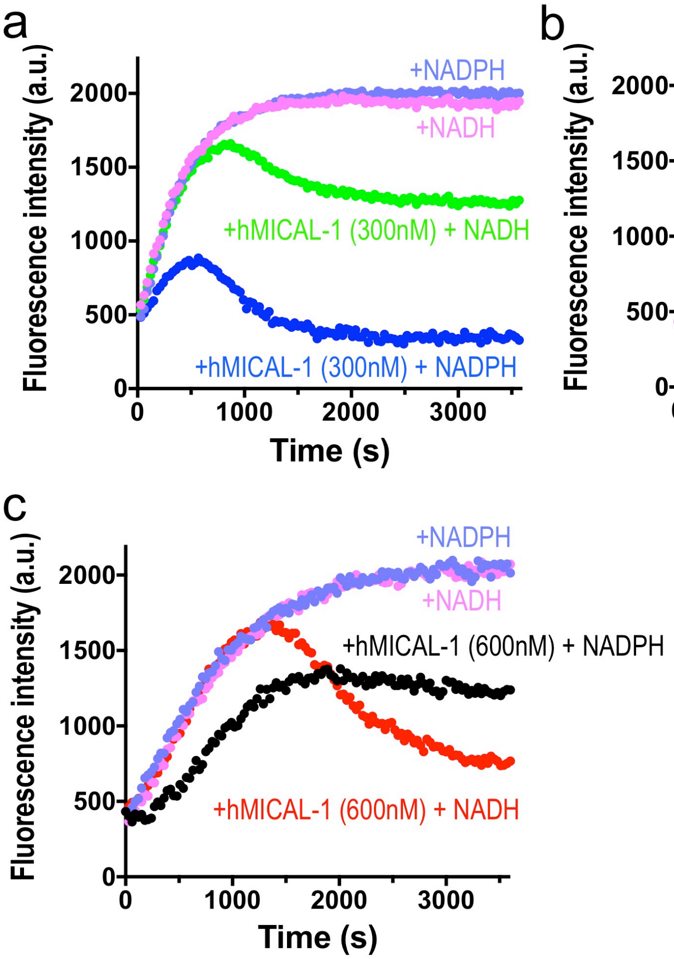

Supplementary Figure 6. Further characterization of human MICALs effects on F-actin dynamics. (a-b) hMICAL-1 (a) and hMICAL-2 and hMICAL-3 (b) alter the rate and extent of both actin polymerization and depolymerization more effectively in the presence of NADPH (100 μM NADPH) than with the related pyridine nucleotide coenzyme NADH (100 μM NADH). [Actin] = 1.15 μM, [MICAL-1]=300 nM, [MICALs-2, 3]=600 nM. **(c)** However, note that NADH can allow higher concentrations of MICAL-1 to more effectively alter F-actin dynamics, since MICAL-1 does not as rapidly consume NADH (see **Figure 1g-h**), and thus can employ it for its oxidation reaction to modify actin. [Actin] = 1.15 μM, [NADH]=100 μM, [NADPH]=100 μM, [MICAL-1]=600 nM.

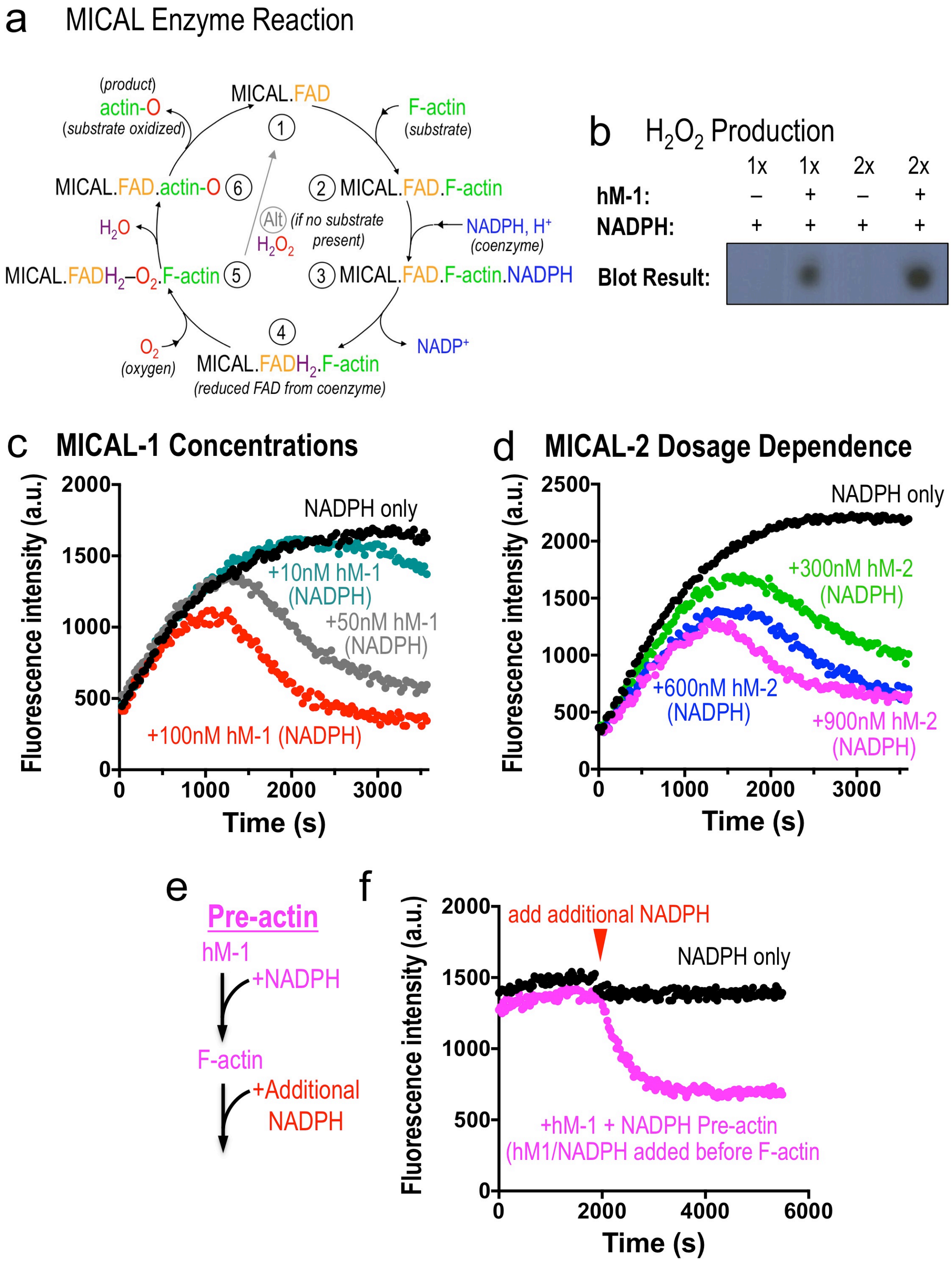

Supplementary Figure 7. MICAL-1 in comparison to other MICALs. (a) Model of the enzyme reaction of the MICALs. MICAL (1) physically associates with its substrate F-actin (2), which triggers MICAL's conversion/consumption of its co-enzyme NADPH to NADP+ (3) and reduction of FAD (4). MICAL then uses oxygen (5), which results in oxidation of F-actin subunits on their Met44 and Met47 residues (6). When MICAL's F-actin substrate is not present, NADPH can be consumed at more limited levels (see **Figure 3a**; [5]) and H_2O_2 can be produced (Alt). **(b)** Further analysis of the generation of hydrogen peroxide by MICALs. Generation of hydrogen peroxide (H_2O_2) by human MICAL-1 (hM-1) as revealed by chemiluminescent detection of H_2O_2 produced by hM-1 in the presence of a substrate (luminol) and different concentrations (1x or 2x the amount) of a catalyst (horseradish peroxidase (HRP)). [MICAL-1]=600nM, [NADPH]=100μM. **(c)**

Pyrene-actin assays reveal that at low concentrations, hMICAL-1's ability to alter actin polymerization is dosage dependent. However, with increasing higher concentrations, MICAL-1 exhibits decreasing effects on F-actin (see **Figure 4d**). These effects are consistent with hMICAL-1 exhibiting such rapid consumption of NADPH in the absence of its F-actin substrate that NADPH becomes limiting (as higher levels of hMICAL-1 are added to the assay) in allowing MICAL-1 to alter F-actin dynamics (see also main text). [Actin] = 1.15 μM, [NADPH]=100 μM. **(d)** hMICAL-2's ability to alter actin polymerization is dosage dependent (as observed using pyrene-actin assays). [Actin] = 1.15 μM, [NADPH]=100 μM. **(e-f)** Further analysis of hMICAL-1's effects on actin dynamics. MICAL-1 does not disassemble F-actin when it is incubated with NADPH prior to the addition of F-actin (see **Figure 4f-g**). These results support that MICAL-1, because of its high-rate of basal activity, consumes/uses-up NADPH prior to the addition of F-actin, such that it can no longer use NADPH in its reaction to modify actin. To further test this hypothesis, we added more NADPH (additional NADPH in e-f) into the MICAL-1 pre-reaction condition tube (f, arrowhead) (from Supplementary Figure 6c). MICAL-1 now proceeds to modify actin and induce F-actin disassembly (f, pink). [Actin] = 1.15 μM, [MICAL-1]=600nM, [NADPH]=100 μM. Unprocessed original scans of blots are shown in Supplementary Fig. 11.

hMICAL-1^{redoxCH} DG mutation purification

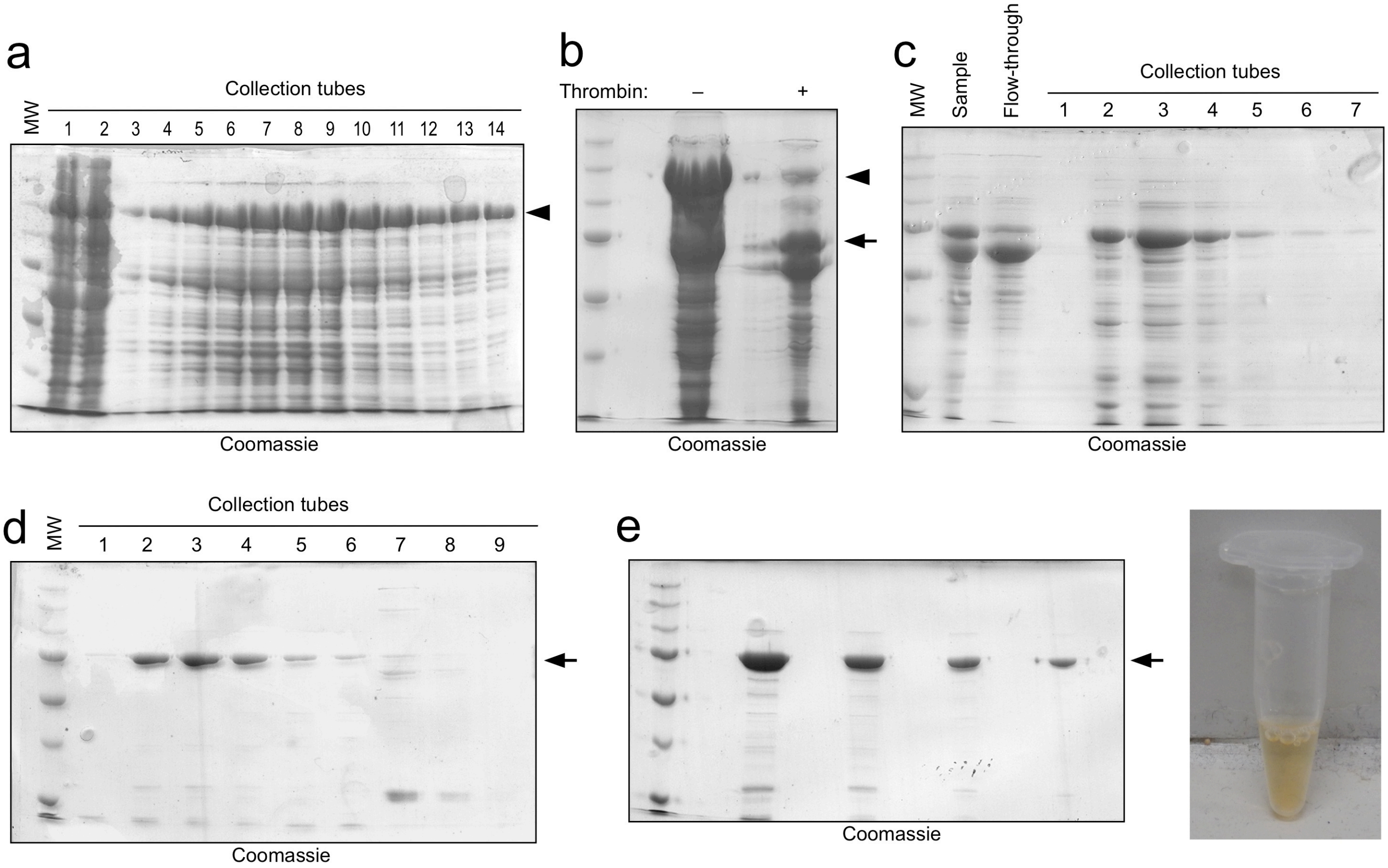

Supplementary Figure 8. Purification and characterization of human MICAL-1^{redoxCH} DG protein (hMICAL-1^{DG}). Compare also with the strategy used in Supplementary Figure 1 to purify human MICAL-1^{redoxCH}. Coomassie stained gels are shown and the arrows point to the recombinant hMICAL-1^{redoxCH} DG protein in all gels. **(a)** A cDNA encoding hMICAL-1^{redoxCH} DG was inserted into a His-tag containing bacterial expression vector, transformed into bacteria, and following the appropriate growth conditions, lysates were loaded on a Ni-NTA affinity column to enrich for the Nus/His-tagged hMICAL-1^{redoxCH} DG (arrowhead). **(b)** The Nus-tagged hMICAL-1^{redoxCH} DG (arrowhead) was digested (+) with a thrombin protease to cleave-off the Nus tag. The smaller size of the digested human MICAL-1^{redoxCH} DG (without the Nus-His tag) can be seen (arrow). **(c)** The digested (+) sample from (b) was then loaded again on a Ni-NTA agarose column to remove the Nus-tag. Fractions from 2-6 were combined and used for d. **(d)** Ion-exchange chromatography was then used to remove contaminating proteins since the hMICAL-1^{redoxCH} DG can bind with the MonoQ column (arrow). Samples within collection tubes 2-6 were then combined and concentrated **(e)** and analyzed on a gel to determine the purity of the hMICAL-1^{redoxCH} DG protein. The purified hMICAL-1^{redoxCH} DG mutant is also shown in a transparent tube, where its yellowish color is readily observed. Note that the yellow color is lighter than the other MICALs (**Supplementary Figures 1-3**), because of more protein being made without FAD bound to it (**Supplementary Figure 4c**).

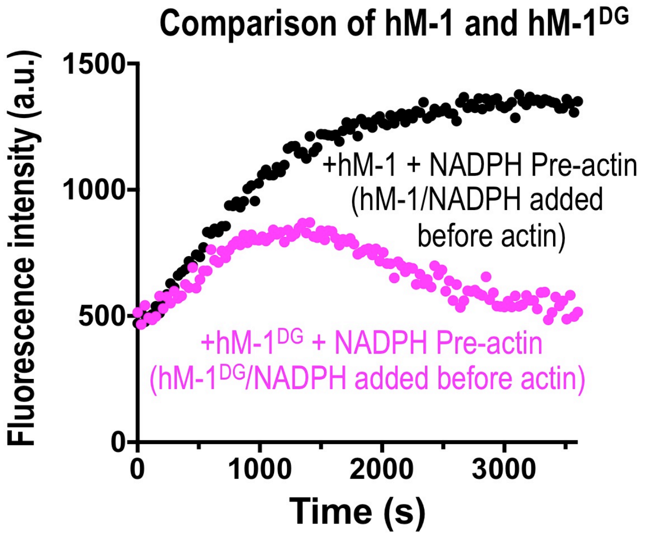

Supplementary Figure 9. Further characterization of human MICAL-1^{DG} (hM-1^{DG}). Using the method outlined in **Figure 4f**, hM-1 or hM-1^{DG} was incubated with NADPH prior to the addition of actin. Then, using a pyrene actin assembly assay as in **Figure 2b**, note that MICAL-1 no longer alters actin polymerization when it is incubated with NADPH prior to the addition of actin (black), whereas making the DG alteration (hM-1^{DG}) enables M-1 to alter actin polymerization. [Actin] = 1.15 μM, [MICAL-1]=600nM, [NADPH]=100 μM.

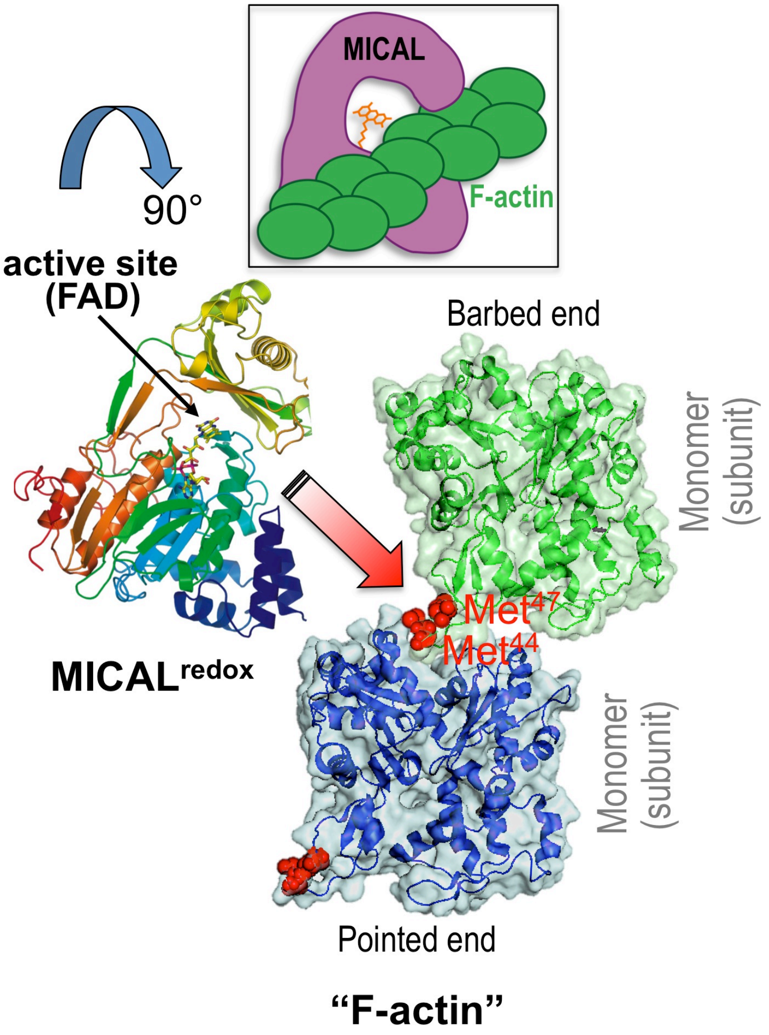

Supplementary Figure 10. Model of the MICALs activation, regulation, and interaction with F-actin – and access to Actin's Met44 and Met47 residues within filaments. The MICALs dismantle actin filaments by oxidizing actin subunits on their Met44 and Met47 residues. Yet, previous work from others have revealed that when actin is present in filaments, the Met44 and Met47 residues of actin are poorly accessible to diffusible solvents, including oxidants such as hydrogen peroxide [6-9]. Therefore, these results suggest the hypothesis that MICALs do not modify F-actin through the general release of hydrogen peroxide or another diffusible oxidant. Likewise, direct experiments also support this hypothesis by revealing that hydrogen peroxide has no effects on F-actin disassembly (even when added at high millimolar concentrations), that hydrogen peroxide does not disassemble F-actin in combination with MICALs binding to F-actin, and that hydrogen peroxide scavengers do not alter MICAL-mediated actin disassembly ([4,5,10-13]; present study). Further, the general release of hydrogen peroxide or another diffusible oxidant would not be expected to modify an amino acid stereospecifically as the MICALs do (which selectively modify actin's Met44 and Met47 residues in a single stereo-specific conformation) – and this modification as well as the MICALs effects on F-actin are selectively reversed by the methionine sulfoxide reductase SelR/MsrB ([10,14]; present study). Moreover, when MICALs are separated from F-actin using a barrier/compartmentalized chamber system, MICALs do not exert effects on F-actin [5]. Thus, all of these results indicate that the active site of the MICALs needs to gain access to the poorly accessible Met44 and Met47 residues that are buried within F-actin. A model is now emerging based on previous work and the experiments conducted herein that the MICALs are under tight regulation: both precisely localized and maintained in an inactive conformation in the cell (i.e., without O_2 consumption/NADPH activity/effects on F-actin/ hydrogen peroxide production, etc.) so as to not dismantle all F-actin structures [4,11,15-17]. MICALs are then

locally activated upon binding to other proteins such as the Semaphorin repellent receptor Plexin and small GTPases like Rab35 [4,11,15-18]. Then, when activated, each member of the MICAL family of proteins binds to actin filaments (as illustrated in the inset). This binding to actin filaments then accelerates the enzymatic activity of each MICAL family member, allowing it to consume more NADPH. In conjunction with this activation of the MICAL enzymes, the active site (small arrow) of MICAL accesses (large arrow) the Met44 (Met^{44}) and Met47 (Met^{47}) residues within filaments. Then, the MICALs selectively oxidize both Met44 and Met47 in the *R* stereospecific pattern (generating actin$^{\text{Met44,47–}R\text{–sulfoxide}}$). In turn, because it is thought that hydrophobic interactions are critical for the binding between F-actin subunits [19-22], this selective oxidation of (placing a charge on) Met44 and Met47 by the MICALs weakens the hydrophobic interactions between F-actin subunits – since Met44 and Met47 lie at the interface between subunits (see diagram) – and achieves controlled disassembly of filaments [5,11,23] (see also Ref. 24 for more detail). This disassembly of actin filaments occurs through both severing and depolymerization [4,5,10,11,24]. PDB IDs are 2BRY, 2ZWH [25,26].

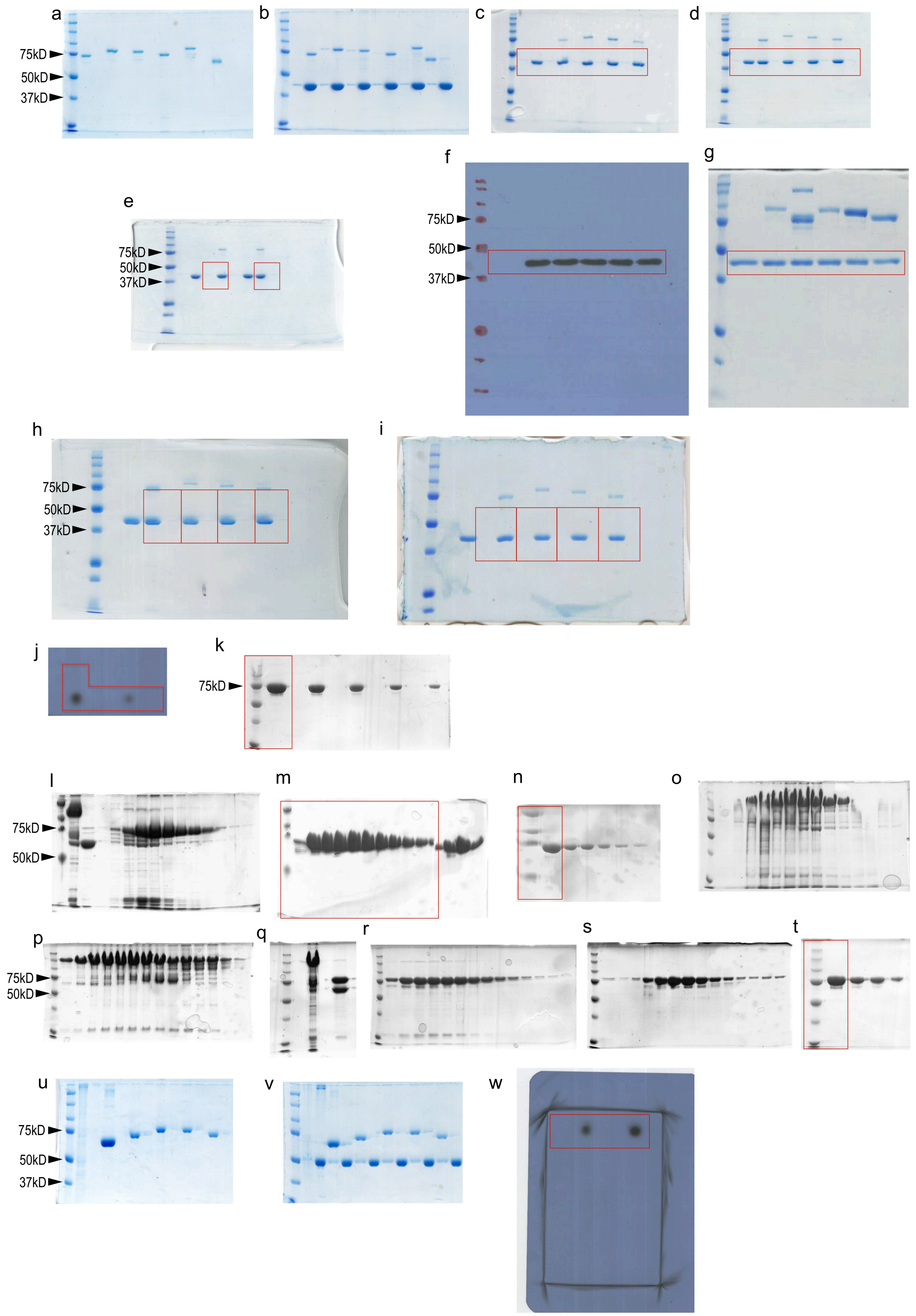

Supplementary Figure 11. Uncropped Gels/Blots. Uncropped gels/blots for Figures 2a Left (a), 2a Right (b), 2c Upper left (c, red box), 2c Lower Left (d, red box), 2c Upper Right and Lower Right (e, left and right red boxes, respectively), 3b Upper (f, red box), 3b Lower (g, red box), 3c WT actin (h, red boxes), 3c M2L actin (i, red boxes), 4b (j, red box), S1e (k, red box), S2c (l), S2d (m, red box), S2e (n, red box), S3a (o), S3b (p), S3c (q), S3d (r), S3e (s), S3f (t, red box), S5a Left (u), S5a Right (v), and S7b (w, red box). All other gels in Figures/Supplementary Figures show the full uncropped gels.

Supplementary References

1 Aliverti, A., Curti, B. & Vanoni, M. A. Identifying and quantitating FAD and FMN in simple and in iron-sulfur-containing flavoproteins. *Methods Mol Biol* **131**, 9-23 (1999).

2 Chapman, S. K. & Reid, G. A. in *Methods in Molecular Biology* Vol. 131 256 (Humana Press, Totowa, NJ, 1999).

3 Wu, H., Hung, R. J. & Terman, J. R. A simple and efficient method for generating high-quality recombinant Mical enzyme for in vitro assays. *Protein Expr Purif* **127**, 116-124 (2016).

4 Hung, R. J. *et al.* Mical links semaphorins to F-actin disassembly. *Nature* **463**, 823-827 (2010).

5 Hung, R. J., Pak, C. W. & Terman, J. R. Direct redox regulation of F-actin assembly and disassembly by Mical. *Science* **334**, 1710-1713 (2011).

6 Dalle-Donne, I. *et al.* Methionine oxidation as a major cause of the functional impairment of oxidized actin. *Free Radic Biol Med* **32**, 927-937 (2002).

7 Guan, J. Q., Almo, S. C., Reisler, E. & Chance, M. R. Structural reorganization of proteins revealed by radiolysis and mass spectrometry: G-actin solution structure is divalent cation dependent. *Biochemistry* **42**, 11992-12000 (2003).

8 Guan, J. Q., Takamoto, K., Almo, S. C., Reisler, E. & Chance, M. R. Structure and dynamics of the actin filament. *Biochemistry* **44**, 3166-3175 (2005).

9 Takamoto, K., Kamal, J. K. & Chance, M. R. Biochemical implications of a three-dimensional model of monomeric actin bound to magnesium-chelated ATP. *Structure* **15**, 39-51 (2007).

10 Hung, R. J., Spaeth, C. S., Yesilyurt, H. G. & Terman, J. R. SelR reverses Mical-mediated oxidation of actin to regulate F-actin dynamics. *Nat Cell Biol* **15**, 1445-1454 (2013).

11 Fremont, S. *et al.* Oxidation of F-actin controls the terminal steps of cytokinesis. *Nat Commun* **8**, 14528 (2017).

12 Hung, R.-J. & Terman, J. R. Extracellular inhibitors, repellents, and Semaphorin/Plexin/MICAL-mediated actin filament disassembly. *Cytoskeleton* **68**, 415-433 (2011).

13 Wilson, C., Terman, J. R., Gonzalez-Billault, C. & Ahmed, G. Actin filaments - a target for redox regulation. *Cytoskeleton (Hoboken)* **73**, 577-595 (2016).

14 Lee, B. C. *et al.* MsrB1 and MICALs Regulate Actin Assembly and Macrophage Function via Reversible Stereoselective Methionine Oxidation. *Mol Cell* **51**, 397-404 (2013).

15 Schmidt, E. F., Shim, S. O. & Strittmatter, S. M. Release of MICAL autoinhibition by semaphorin-plexin signaling promotes interaction with collapsin response mediator protein. *J Neurosci* **28**, 2287-2297 (2008).

16 Giridharan, S. S., Rohn, J. L., Naslavsky, N. & Caplan, S. Differential regulation of actin microfilaments by human MICAL proteins. *J Cell Sci* **125**, 614-624 (2012).

17 Vitali, T., Maffioli, E., Tedeschi, G. & Vanoni, M. A. Properties and catalytic activities of MICAL1, the flavoenzyme involved in cytoskeleton dynamics, and modulation by its CH, LIM and C-terminal domains. *Arch Biochem Biophys* **593**, 24-37 (2016).

18 Terman, J. R., Mao, T., Pasterkamp, R. J., Yu, H. H. & Kolodkin, A. L. MICALs, a family of conserved flavoprotein oxidoreductases, function in plexin-mediated axonal repulsion. *Cell* **109**, 887-900 (2002).

19 Holmes, K. C., Popp, D., Gebhard, W. & Kabsch, W. Atomic model of the actin filament. *Nature* **347**, 44-49 (1990).

20 Oda, T., Iwasa, M., Aihara, T., Maeda, Y. & Narita, A. The nature of the globular- to fibrous-actin transition. *Nature* **457**, 441-445 (2009).

21 Galkin, V. E., Orlova, A., Vos, M. R., Schroder, G. F. & Egelman, E. H. Near-atomic resolution for one state of F-actin. *Structure* **23**, 173-182 (2015).

22 von der Ecken, J. *et al.* Structure of the F-actin-tropomyosin complex. *Nature* **519**, 114-117 (2015).

23 Grintsevich, E. E. *et al.* F-actin dismantling through a redox-driven synergy between Mical and cofilin. *Nat Cell Biol* **18**, 876-885 (2016).

24 Grintsevich, E. E. *et al.* Catastrophic disassembly of actin filaments via Mical-mediated oxidation, *in press*.

25 Siebold, C. *et al.* High-resolution structure of the catalytic region of MICAL (molecule interacting with CasL), a multidomain flavoenzyme-signaling molecule. *Proc Natl Acad Sci U S A* **102**, 16836-16841 (2005).
26 Nadella, M., Bianchet, M. A., Gabelli, S. B., Barrila, J. & Amzel, L. M. Structure and activity of the axon guidance protein MICAL. *Proc Natl Acad Sci U S A* **102**, 16830-16835 (2005).
